# Identification of Potential Cytochrome P450 3A5 Inhibitors: An Extensive Virtual Screening through Molecular Docking, Negative Image-Based Screening, Machine Learning and Molecular Dynamics Simulation Studies

**DOI:** 10.3390/ijms23169374

**Published:** 2022-08-19

**Authors:** Md Ataul Islam, Dawood Babu Dudekula, V. P. Subramanyam Rallabandi, Sridhar Srinivasan, Sathishkumar Natarajan, Hoyong Chung, Junhyung Park

**Affiliations:** 1 3BIGS Omicscore Private Limited, 909 Lavelle Building, Richmond Circle, Bangalore 560025, India; 2 3BIGS Co., Ltd., B-831, Geumgang Penterium IX Tower, Hwaseong 18469, Korea

**Keywords:** cytochrome P450 3A5, cardiovascular diseases, virtual screening, molecular docking, negative image-based screening, machine learning

## Abstract

Cytochrome P450 3A5 (CYP3A5) is one of the crucial CYP family members and has already proven to be an important drug target for cardiovascular diseases. In the current study, the PubChem database was screened through molecular docking and high-affinity molecules were adopted for further assessment. A negative image-based (NIB) model was used for a similarity search by considering the complementary shape and electrostatics of the target and small molecules. Further, the molecules were segregated into active and inactive groups through six machine learning (ML) matrices. The active molecules found in each ML model were used for in silico pharmacokinetics and toxicity assessments. A total of five molecules followed the acceptable pharmacokinetics and toxicity profiles. Several potential binding interactions between the proposed molecules and CYP3A5 were observed. The dynamic behavior of the selected molecules in the CYP3A5 was explored through a molecular dynamics (MD) simulation study. Several parameters obtained from the MD simulation trajectory explained the stability of the protein–ligand complexes in dynamic states. The high binding affinity of each molecule was revealed by the binding free energy calculation through the MM-GBSA methods. Therefore, it can be concluded that the proposed molecules might be potential CYP3A5 molecules for therapeutic application in cardiovascular diseases subjected to in vitro/in vivo validations.

## 1. Introduction

Over the last few decades, the screening of molecular databases has become a fascinating drug-discovery strategy in academia and industry [1,2]. The screening of large chemical databases is an effective and pioneering methodology for finding potential molecules or molecular scaffolds for any specific disease target. The computational screening i.e., or virtual screening (VS) of small molecules has gained extreme popularity due to its high efficiency and accuracy along with its low expense and fewer required animal sacrifices [3,4]. Moreover, obtaining effective, potent, and safer drug-like molecules is a cost-intensive, extremely difficult, complex, and extensive trial-and-error process [5]. The VS method incorporates the help of excellent computational power and storage capacity and it can predict and identify potential lead-like molecules within a shorter time span and at an exceedingly low cost [6]. The beauty of the VS method is that it can screen the smaller dataset to ultra-large chemical libraries through any computational model. In the execution of VS, there is no required physical presence of chemical compounds, and it can be evaluated computationally until it enters the synthesis phase. VS can be categorized into ligand- and structure-based methods. In the ligand-based VS (LBVS) method, statistically robust and accurately predictive models are generated from a known set of small molecules together with their biological activities followed by the screening of chemical databases through a set of well-defined criteria [7]. In particular, LBVS identifies and optimizes the small molecules based on the information available in the known active molecules irrespective of the receptor molecule. On the other hand, the structure-based VS (SBVS) method [8] uses the target cavity information and predicts the best interactions between the receptor and ligands followed by the ranking of the molecules according to their binding affinities. The SBVS method requires the three-dimensional (3D) coordinates of the target molecule along with small molecular datasets from public or commercially available databases. Molecular docking has become the routine prototypic methodology of SBVS due to its lower computational expense and effective results [9]. The optimal choice is molecular docking-based screening due to its better understanding of the binding modes of the small molecules inside the receptor cavity of the protein along with its prediction of binding affinity depending upon conformational analyses. A number of well explored commercial and public molecular docking engines are available, which include Glide [10], LigandFit [11], FlexX [12], Gold [13], MOE [14], Dock [11], Autodock (AD) [15], Autodock vina (ADV) [16], etc. The quality and trustworthiness of any docking program are mainly decided by the efficiency of the conformational searching algorithm and the quality of its scoring function (SF) [17]. Without any doubt, the ADV is the most widely used publicly available docking engine for the scientific community worldwide for its speed and reliability [18]. Negative image-based (NIB) [19] screening is a another crucial approach to screening large chemical datasets. This method comprises the 3D structure of the target molecule bound with a ligand in the receptor cavity. The NIB model uses the shape and electrostatic complementarity of the receptors to the ligands to screen more potential molecules. The application of machine learning (ML) methods in drug-discovery research opens up a wider window to identify active chemical compounds with less effort and within a shorter amount of time. Hence, the application of the above computational methodologies to any certain target will be an effective and efficient approach to finding promising chemical entities.

Cytochrome P450 (CYP) is a well-known and well-studied hemeprotein that functions in the metabolism of drugs and foreign substances [20]. It plays the roles of inhibitors, inducers, or substrates for the different enzymatic pathways and for modifying the metabolic pathways of the co-administered drugs [21]. About three-quarters of marketed drugs are metabolized by CYP enzymes, and the CYP3A family alone metabolizes one-half of those drugs [22,23]. In particular, from 2013 to 2017, it was estimated that more than 65% of Food and Drug Administration- (FDA) approved drugs were CYP3A substrates [21]. Out of the four members of the CYP3A family, namely, CYP3A4, CYP3A5, CYP3A7, and CYP3A43, only CYP3A4 and CYP3A5 are abundantly expressed in adults [24]. A vast amount of research has illustrated that CYP3A5 shows metabolic activity towards many drugs, including tacrolimus, vincristine, and maraviroc [25,26,27]. Moreover, regarding tacrolimus, an approved drug used for heart and liver transplants, it was shown that CYP3A5 clears the drug faster than CYP3A4 [28,29]. A polymorphism of CYP3A5 showed different reactivity towards several hypertensive drugs, thus affecting the blood pressure in the studied individuals [30]. Several studies have already used CYP3A5 as a potential target for the in silico assessment of potential molecules [31,32,33,34]. Hence, the above data and research findings substantiated that CYP3A5 is a crucial and validated drug target for cardiovascular and other diseases [35]. Overall, the main objective of the current study was to screen the large chemical database against the CYP3A5 target for cardiovascular diseases through molecular docking, NIB screening, ML-based screening followed by pharmacokinetics and a toxicity assessment, molecular dynamics (MD) simulation, and molecular mechanics generalized Born surface area- (MM-GBSA) based binding free energy calculation. The validity of the work was substantiated via the identification of five high-affinity promising inhibitor molecules targeting CYP3A5.

## 2. Results and Discussion

### 2.1. Virtual Screening

**Figure 1:**
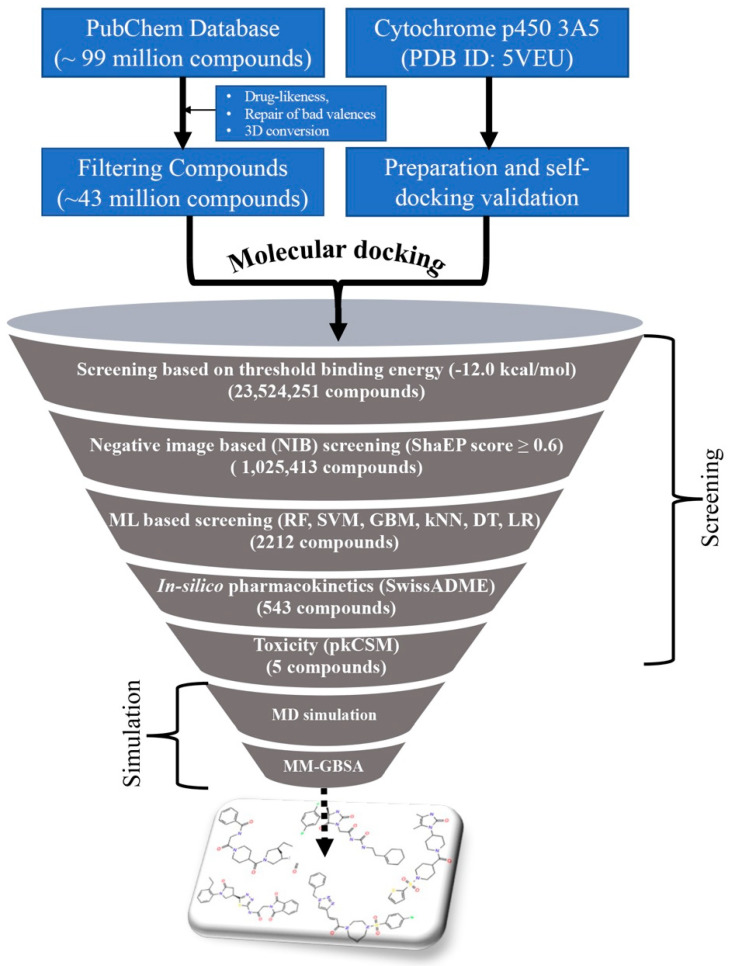
The workflow of screening the PubChem database against CYP3A5.

The entire PubChem database was curated with several screening criteria and narrowed down to about 44 million molecules. The curated PubChem database was screened through a number of advances and conventional cheminformatics approaches such as molecular docking, NIB screening, ML method, in silico pharmacokinetics, and toxicity evaluation to identify promising and potential CYP3A5 compounds. The stepwise flow diagram of the entire work scheme employed is given in Figure 1.

#### 2.1.1. Molecular Docking

Molecular docking is an excellent computational method for screening a vast chemical library dataset to identify prospective chemical entities that could be used to explore the binding potentiality for a specific target. In the last two decades, molecular docking has become the prototype of structure-based screening of various in-house created chemical libraries or virtually available chemical databases [9]. In a similar fashion, in the present study, molecular docking was performed for the curated PubChem database against the CYP3A5 protein. However, before the docking was carried out for the entire curated PubChem database, the employed molecular docking methodology was validated using the self-docking approach. Therefore, the co-crystallized small molecule (RIT) was drawn and re-docked at the same location where the co-crystal ligand was originally bound with CYP3A5. After re-docking, the best binding pose was superimposed on the original co-crystal ligand and the root-mean square deviation (RMSD) was found to be 1.913 Å after superimposition, indicating the successful validation of the docking protocol. The superimposed orientation of the re-docked and original co-crystal conformer is given in Figure S1 (Supplementary Data). The active site coordinates and grid box size utilized in the validated self-docking approach were further considered for the molecular docking of the PubChem molecules. The ADV [16] program, a publicly available docking tool, was used to execute the molecular docking study of all the compounds under study. On the default parameter setting, this freely available tool provides the nine best dock poses, and they are ranked according to their binding affinity scores. A higher negative binding affinity suggested the better binding potentiality of any small molecule towards the target molecule. After successful docking, the binding affinity or energy score (in kcal/mol) of the best-docked pose of each molecule was explored. The binding affinity of all the docked molecules was found to be within the range of −1.04 to −15.23 kcal/mol. Further, to narrow down the chemical space, the user-defined binding energy threshold was −12.00 kcal/mol, i.e., molecules with a binding energy equal to or less than the threshold were selected for further assessment. It was observed that more than 23.5 million compounds were found to have a binding energy less than or equal to −12.00 kcal/mol toward the CYP3A5. All these molecules were further screened through the NIB screening approach.

#### 2.1.2. Negative Image-Based (NIB) Screening

**Figure 2:**
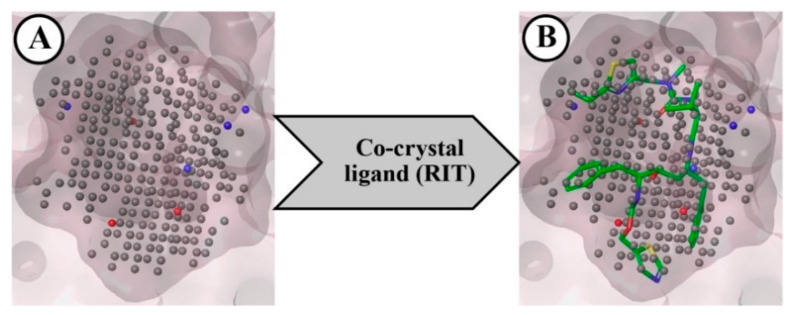
Negative-image based (NIB) model developed from CYP3A5 receptor cavity (**A**), and RIT model mapped with NIB model (**B**).

The NIB model was created by considering the geometry and electrostatic properties of the ligand-binding receptor cavity of CYP3A5. This model mainly focuses on the major components of the shape features that are crucial for the successful binding of the ligand to CYP3A5’s key amino acid residues at the active site. The final NIB model obtained for the CYP3A5 is given in Figure 2A. The final model was found to have several important cavity points represented by negative (red), positive (blue), and neutral (grey) charge/features for the effective binding of the potential ligand. The co-crystal ligand (RIT) was fitted to the NIB model, and it is portrayed in Figure 2B. The ShaEP score of RIT was found to be 0.600 and it was considered the threshold score for the further screening of the docked PubChem molecules.

**Figure 3:**
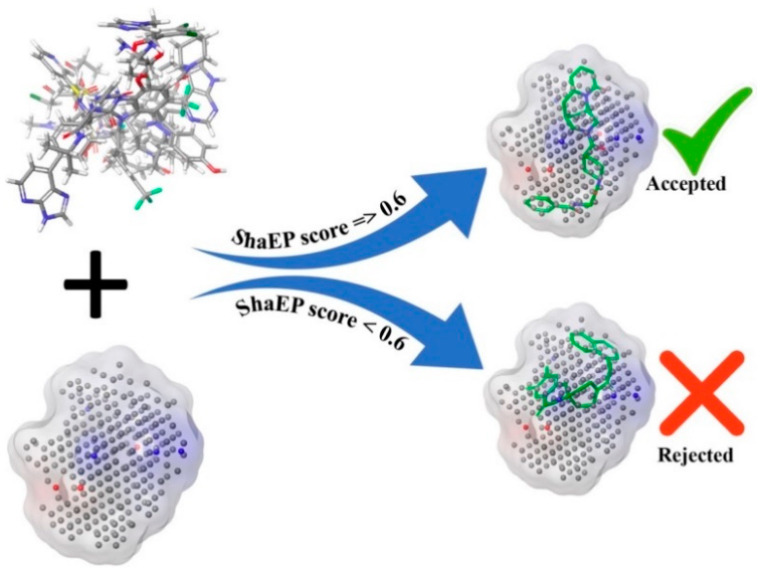
Screening workflow of PubChem docked molecules through ShaEP score of NIB model.

The ShaEP [36] score is the similarity score that extends from 0 to 1, indicating a range from a dissimilar to an exactly similar molecular structure. The molecules retained after the binding energy-based screening were further screened using the ShaEP tool followed by calculating the ShaEP score. The workflow of the ShaEP-based screening is given in Figure 3. The ShaEP score was found to be in the range of 0.255 to 0.771. The threshold ShaEP value (≥0.6) was used to screen the molecules and a total of 1025413 molecules were retained in this step. The abovementioned compounds were further taken into account for an ML-based chemical space reduction.

#### 2.1.3. Machine Learning-(ML) Based Screening

The ML approach is extensively used in drug discovery to explore potential lead-like molecules for any specific target. The molecules retained after NIB screening are considered to segregate into active and inactive by using several ML metrics. In particular, the ML models were utilized to classify the active and inactive molecules from the unknown dataset based on the information from known active and inactive molecular datasets. Initially, a set of known CYP3A5 active molecules with a total of 16 molecules was collected from BindingDB [37]. The low inhibitory activity (IC_50_ < 10 nM) was used to identify the active molecules. By considering the above set of active molecules, a set of decoy molecules (6341) was generated using the DUD-E decoy generator [38]. The amalgamated active and decoy sets were considered as the training set, and the molecules found in the NIB screening were taken as the test set. Both the training and test sets were used to generate the 2D and 3D PaDEL [39] descriptors. In the training set descriptors, a total of 492 features were found significant out of the 1144 PaDEL descriptors using Wilcoxon’s rank-sum test with the significance of *p* < 0.05. The above features were further used in the scikit-learn package in Python 3.6 for the ML models’ development. A total of six different ML models, including decision trees (DT) [40], logistic regression (LR) [41], *k*-nearest neighbor (*k*-NN) [42], random forest (RF) [43], support vector machines (SVM) [44], and gradient-boosting machines (GBM) [45] were generated. Prior to the use of ML models in the test set, each model was validated by McNemer’s test, which found no significant difference between the training set and the ML models. Several model performance measures were calculated, including precision, recall, F-score, accuracy, and specificity from the confusion matrix (CM) for all the six ML models, and these measures are given in Table 1. The fivefold cross-validation results for all the six models explain the statistical robustness of the models.

**Figure 4:**
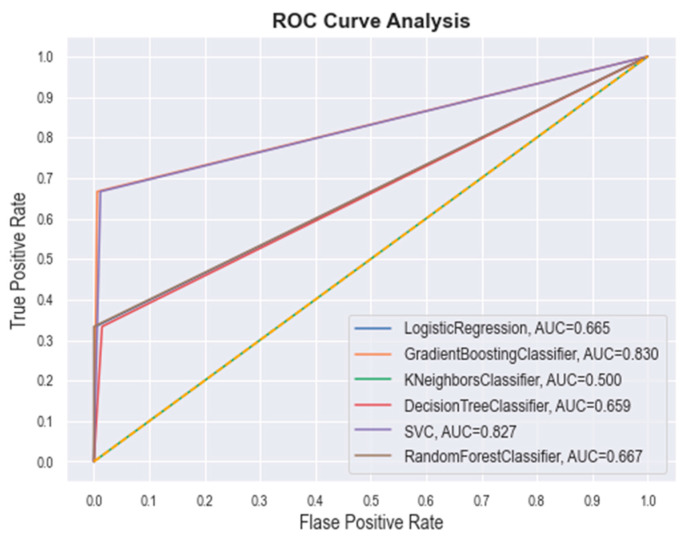
ROC curves for different ML classifiers.

The best hyperparameter classifiers for SVM were determined to be C = 1.0, kernel = ‘linear’, gamma = 0.002, and class_weight = ‘balanced’. In the case of the best RF hyperparameters, the classifiers were noted as n_estimators = 100, criterion = ‘gini’, min_samples_split = 2, min_samples_leaf = 1, class_weight = ‘balanced’, and min_ weight_fraction_leaf = 0.0. For the GBM, the revealed classifiers were loss = ‘log_loss’, learning_rate = 0.2, n_estimators = 100, criterion = ‘friedman_mse’, min_samples_split = 2, and min_samples_leaf = 1. The DT classifiers were found to be criterion = ‘gini’, min_samples_split = 2, min_samples_leaf = 1, min_weight_ fraction_leaf = 0.0, and class_weight = ‘balanced’. The LR classifiers were found to be penalty= ‘l2’, dual = False, C = 1.0, fit_intercept = True, intercept_scaling = 1, solver = ‘lbfgs’, max_iter = 100, and class_weight = ‘balanced’. Finally, the KNN classifiers were found to be n_neighbors = 3 and weights = ‘distance’. Further, the quality of each model was checked through the receiver operating characteristic (ROC) plot, which represents the false positive rate (x-axis) and the true positive rate (y-axis) for all the samples with a threshold between 0 and 1. The ROC curve of each model was plotted and are given in Figure 4. The area under curve (AUC) value of GBM, SVM, RF, LR, DT, and *k*-NN was found to be 0.830, 0.827, 0.667, 0.665, 0.659, and 0.500, respectively. The above obtained data certainly suggest the potential of each ML model.

The test set molecules were considered for segregation into active and inactive compounds using the developed six ML models. Based on their significant features, each ML model identifies the active and inactive compounds from the given input test set. After the successful screening of the test set, active molecules from each model were retained for further assessment. The duplicate molecules from the amalgamated active compounds were deleted. Finally, a total of 2212 unique active compounds remained in the dataset for further assessment.

#### 2.1.4. In Silico Pharmacokinetics and Toxicity-Based Screening

**Figure 5:**
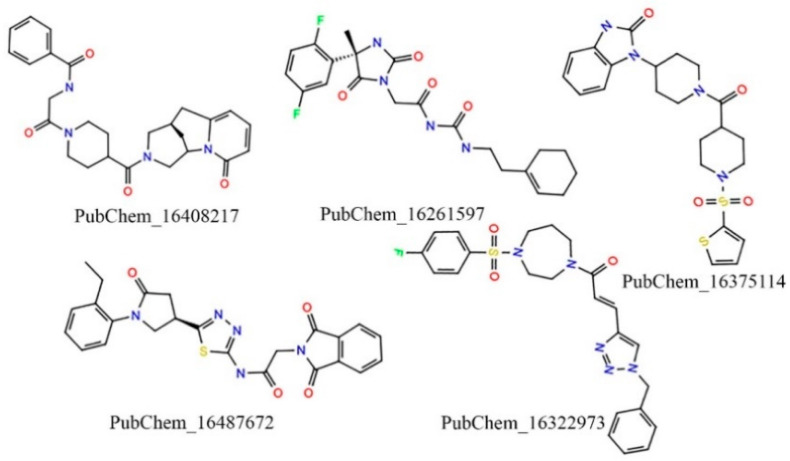
Two-dimensional (2D) representation of the final promising proposed CYP3A5 molecules.

The molecules retained after the ML-based screening were considered for the in silico pharmacokinetics and toxicity assessments. Various pharmacokinetics and toxicity parameters were calculated using two well-known ADMET prediction programs: SwissADME [46] and pKCSM [47], respectively. Several pharmacokinetic profiles, including hydrophobicity (logP), solubility, GI and BBB absorption, and synthetic accessibility, were considered as the filtration criteria to reduce the chemical space. Particularly, the accepted parameters were defined as logP ≤ 5, a degree of solubility that was either very soluble or soluble, GI absorption = yes, BBB absorption = no, and synthetic accessibility ≤ 6 to screen the potential drug-like candidate molecules. With respect to the above criteria, 543 molecules were retained. Further, the molecules that fulfilled the pharmacokinetic assessment were further evaluated for the toxicity prediction. Several toxicity parameters were calculated for all retained molecules. Herein, the molecules were screened based on AMES toxicity = no, maximum tolerated dose (human) < 4, oral rat toxicity (LD_50_) < 3.2, and skin sensitivity = no. After implementing the abovementioned screening criterion, a total of five of the highest potential drug-like candidate molecules were retained. A two-dimensional (2D) chemical representation of the finally retained molecules is given in Figure 5.

Overall, the exhaustive and systematic approaches—such as employing molecular docking, NIB based screening, advanced ML model-based segregation into active and inactive, and pharmacokinetic and ADMET assessments—applied to the abovementioned five molecules (PubChem_16408217, PubChem_16261597, PubChem_16375114, PubChem_16487672, and PubChem_16322973) were found to be crucial for the successful inhibition of CYP3A5. Upon close observation, it was revealed that several functional groups, including oxo, amine, imine, halogen, non-aromatic and aromatic rings, sulfanyl, etc., are present in the finalized molecules. These critical functional groups might be very important components for binding interactions with key amino acids of CYP3A5 at the active site to hold the proposed molecules in an active conformation to trigger desirable biological effects. The docking-based binding energy of the standard compound RIT was found to be −9.90 kcal/mol, whereas for the proposed molecules the binding energies were observed between −12.10 to −13.60 kcal/mol. In particular, during docking the binding energies of PubChem_16408217, PubChem_16261597, PubChem_16375114, PubChem_16487672, and PubChem_16322973 were found to be −13.00, −12.20, 12.50, 13.60, and −12.70 kcal/mol, respectively. A high negative binding energy in comparison to RIT was the substantiated evidence in favor of the high binding affinity of the proposed molecules toward CYP3A5.

### 2.2. Binding Interaction Analysis

CYP3A5 is a HEME-containing transmembrane protein and its presence plays a crucial role in binding small molecules in the active site. The retrieved CYP3A5 crystal structure was used to obtain the original binding interactions between the various amino acids and the HEME molecule. The binding interaction profile of a HEME molecule is given in Figure S2 (Supplementary Data). It is evident that the metal iron (Fe) atom present at the center of the HEME formed a metallic bond with Cys441. Several HB and hydrophobic interactions, as well as salt bridges, were seen to develop between the HEME and CYP3A5 amino acid residues. The only amino acid residue, Thr436 of CYP3A5, established a HB with HEME. Ile118, Ile184, Ile301, Phe302, Ala305, Val313, Leu364, Val369, Ala370, Phe434, Ile442, and Phe446 were found to be critical for establishing hydrophobic contacts with HEME. Arg105, Arg130, Arg375, and Arg439 were revealed as important amino acids for forming salt bridges with HEME. Since in the molecular docking the position and binding interaction profile of HEME was intact, in the subsequent section, only the binding interaction profile of the final molecules with the amino acid residues of CYP3A5 are discussed.

**Figure 6:**
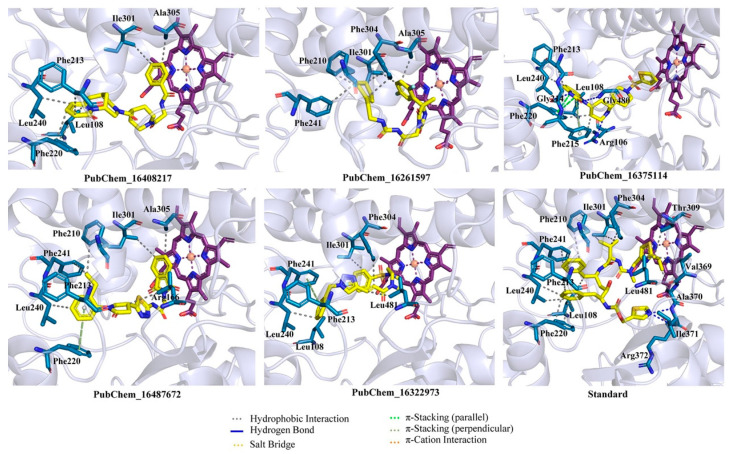
Binding interaction profile of final CYP3A5 molecules. A number of hydrogen and hydrophobic binding interactions are observed.

The binding interaction profile of the final five best molecules for the CYP3A5 was explored and it is given in Figure 6. The binding interactions between RIT and CYP3A5 were explored, and it was found that the Ile371 and Arg372 of CYP3A5 established HB interactions with RIT. Each of the residues Leu108, Phe220, Leu240, Phe241, and Ile301 formed two hydrophobic contacts with RIT. Beyond the above, Phe201, Phe213, Phe304, Thr309, Val369, Ala370, and Leu481 formed a single hydrophobic contact with the RIT.

The final proposed hit molecules were found to interact with CYP3A5 through several crucial HBs and hydrophobic contacts. The terminal phenyl ring of the PubChem_16408217 was found to be very important for forming two hydrophobic contacts with Ile301 and Ala305 of CYP3A5. The presence of a pyridinone ring in PubChem_16408217 was revealed to be crucial for the creation of hydrophobic interactions with Phe213, Phe220, and Leu240. Leu108 of CYP3A5 also formed hydrophobic contact with the octagonal ring of the PubChem_16408217. Another proposed molecule, PubChem_16261597, formed intermolecular interactions at the active site cavity of CYP3A5 by means of hydrophobic contacts between amino acid residues Ile301 and Ala305 of CYP3A5 and the di-fluro phenyl ring of PubChem_16261597. The cyclohexene ring present in PubChem_16261597 was found to be important for imparting the hydrophobicity of the molecule and this was corroborated by the formation of a number of hydrophobic contacts with different amino acids of CYP3A5. Each of the Ile301 and Phe304 amino acids formed two hydrophobic contacts with cyclohexene of PubChem_16261597. Moreover, other amino acid residues, such as Phe210 and Phe241 of CYP3A, also generated one hydrophobic contact with the same group of PubChem_1626159, observed in the molecular docking analysis.

The 2-benzimidazolinone moiety present in PubChem_16375114 was found to be crucial for forming HBs and hydrophobic and π-stacking interactions with several amino acid residues of CYP3A5. Particularly, the nitrogen atom and -oxo group present in benzimidazolinone formed critical HB interactions with Phe215 and Gly480, respectively. Another -oxo group present in the connection between the two piperidine rings in PubChem_16375114 was found to establish the HB interaction with residue Arg106. Three amino acids of CYP3A5, namely, Leu108, Phe213 and Leu240, were successfully connected separately with PubChem_16375114 through a single hydrophobic interaction. Both rings of benzimidazolinone also established π-stacking interactions with residues Phe215 and Phe220. PubChem_16487672 was found to interact through two types of molecular interactions with CYP3A5: hydrophobic and π-stacking. The separate phenyl ring present in PubChem_16487672 was critically stacked with one hydrophobic contact with each of the residues Phe213 and Leu240, along with π-stacking interactions with residue Phe220 of CYP3A5. It was observed that the ethyl group attached to the phenyl ring interacted with two amino acids, namely, Phe210 and Phe241 of CYP3A5. The six-membered ring of the phthalimide in PubChem_16487672 was crucially revealed as important for hydrophobicity and established hydrophobic contacts with residues Ile301 and Ala305. Beyond that, the pyrrolidone also formed hydrophobic contact with Leu481. The presence of a phenyl ring in PubChem_16322973 accounted for a number of hydrophobic and π-stacking interactions. The abovementioned phenyl ring was also involved in the creation of hydrophobic interactions with each of the residues Leu108, Phe213, and Leu240, along with a π-stacking interaction with Phe241. It was observed that the fluro-benzene ring of PubChem_16322973 formed hydrophobic contact with Ile301. Moreover, the alkene part of the molecule was also found to be crucial for forming two hydrophobic interactions with Phe304 and Leu481.

**Figure 7:**
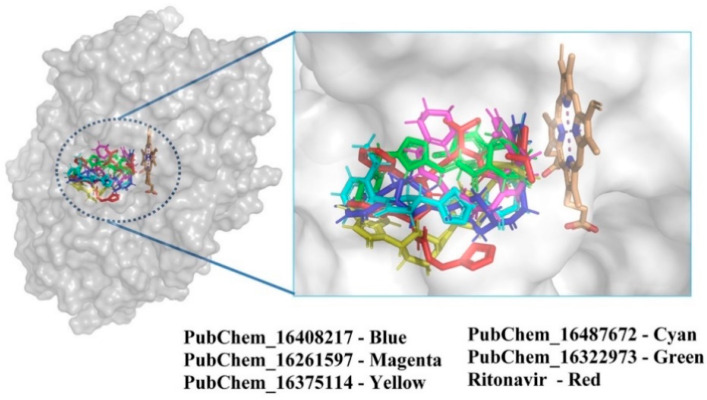
Position of CYP3A5 molecules inside the receptor cavity; a surface view representation.

The best-docked conformer of each proposed compound was explored in the 3D representation to obtain a better insight into the binding orientation at the active site cavity of the CYP3A5 protein; therefore, the surface view was obtained and depicted in Figure 7. Interestingly, it is evident that all the proposed molecules and the standard compound RIT were perfectly fitted into the same position on the active site of CYP3A5. A number of studies reported the molecular docking of small molecules at the active site of CYP3A5. Wu et al. [31] performed the molecular docking of 12 small molecules to CYP3A5, and Phe210 was found to be a critical amino acid for the binding interaction. In the current study, both PubChem_16261597 and PubChem_16487672 along with RIT were found to interact with Phe210. In another study, Saba et al. [48] explored the comparative MD simulation and binding interaction analyses of vincristine in CYP3A4 and CYP3A5. They have reported several ligand-binding amino acids. Among them, Arg106, Leu108, Phe213, Gly214, Phe304, and Gly480 were found to be important ligand-binding amino acids, as in the current study. Dai et al. [34] explored the binding interaction analysis of bufotalin with CYP3A5 and found Arg105, Arg106, and Glu374 to be important amino acids. In the current work, Arg106 was found to be critical for forming the binding interactions with the proposed CYP3A5 molecules. Consequently, it was undoubtedly clear that the binding interactions of the proposed molecules with CYP3A5 were substantiated by the previously published literature. Hence, the analyses and presentation of the binding interactions of the molecules inside the receptor cavity certainly explained the potentiality of the molecules. Most of the binding interactions were observed between the functional groups of the proposed molecules and the ligand-binding amino acids of CYP3A5. Hydrophobic, HB, and π-stacking interactions were found to be key molecular interactions for almost all the molecules. The abovementioned molecular interactions between the CYP3A5 and proposed molecules may be a critical factor for maintaining the protein–ligand complex’s stability.

### 2.3. Mapping of Proposed Molecules on NIB Model

**Figure 8:**
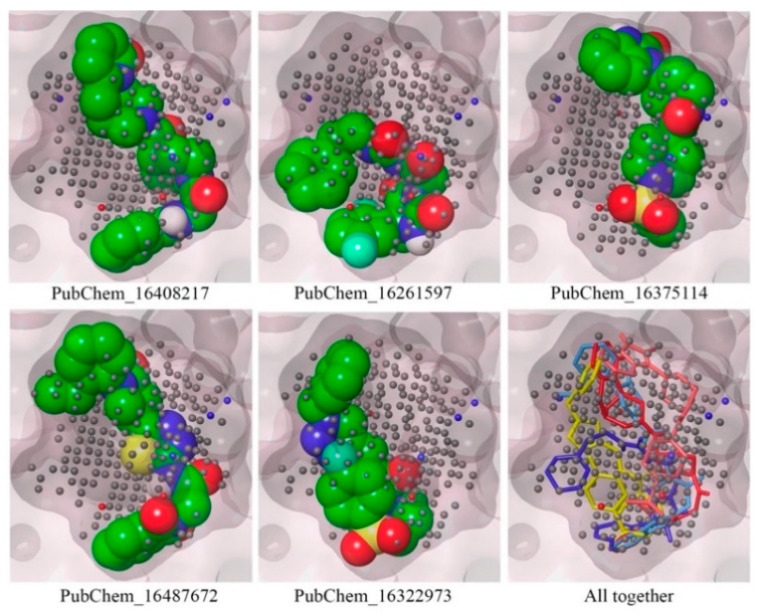
Mapping of final proposed CYP3A5 molecules on the NIB model.

To confirm the mapping orientation in the cavity locations or points, each of the proposed molecules was mapped in the NIB model. The molecules mapped in the NIB model are given in Figure 8. All the molecules were correctly aligned or mapped with the relevant cavity sites of the NIB model, as shown in Figure 8. The ShaEP scores of PubChem_16408217, PubChem_16261597, PubChem_16375114, PubChem_16487672, and PubChem_16322973 were found to be 0.721, 0.695, 0.749, 0.731, and 0.715, respectively. It is worth noting that not a single molecule was found outside the cavity or beyond the cavity points. Hence, the above observations certainly illustrate that the orientation of the final molecules may be an active conformer for the successful inhibition of CYP3A5.

### 2.4. In Silico Pharmacokinetic and Drug-Likeness Assessment

A number of pharmacokinetic and drug-likeness parameters of the final proposed molecules were calculated and estimated; the results are given in Table 2. All the molecules were found to be soluble in nature and highly absorbable in the GI tract. Moreover, not a single molecule was found to penetrate through the brain, i.e., they were non-absorbent through the BBB. The synthetic accessibility explains the ease of the molecule’s synthesis. The synthetic accessibility value of less than six clearly explained that all the molecules were easily synthesizable. It is reported that for a drug-like molecule, the number of heavy atoms (HA) should be between 20 to 70 [49]. The obtained results indicate that the number of HA in all the molecules were within the accepted range. The presence of aromatic rings in any molecule is also crucial to its ability to behave as a lead-like molecule [50]. The presence of at least six aromatic heavy atoms (AHA) in the molecules clearly explains the lead-like behavior of the proposed molecules. The rotatable bonds (RB) of any molecule explain its rigidity or flexibility. None of the molecules were found to have more than eight RB, which shows that these molecules are not very flexible in nature. To form the HB interactions with the amino acid residues of the target molecule, the presence of an HB acceptor (HBA) and an HB donor (HBD) in any small molecule is essential. From Table 2, it can be observed that at least four HBAs and one HBD are present in all the molecules except PubChem_16322973. The molar refractivity (MR) value of all the molecules was found to be less than 134, which also suggests their drug-like characteristics [49]. The total polar surface area (TPSA) within the range of 140 also reflects the lead similarity of the proposed molecule. Hence, from the above discussion and the obtained calculated data, it is quite clear that the proposed molecules were pharmacokinetically sound and possess a good drug similarity profile.

### 2.5. Toxicity Assessment

The toxicity of the proposed molecules was explored, and the results are given in Table 3. The mutagenicity of any molecule can be predicted through the AMES toxicity parameter. Not a single proposed compound was found to have a positive AMES toxicity. This suggests that all the final proposed molecules were non-mutagenic and non-carcinogenic. The lethal dose at the 50% level was explored using the oral rat acute toxicity (ORAT) property. The low ORAT value (<0.4) undoubtedly explained the low acute toxicity of the proposed compounds. The minnow toxicity is another important toxicity-assessment parameter that represents the concentration of a molecule necessary to cause the death of 50% of Flathead minnows. It is reported that a value of the minnow toxicity of less than 0.3 is highly toxic.

The data in Table 3 suggest that none of the proposed molecules are acutely toxic in nature. The maximum tolerated dose (MTD) is a prediction of the toxic dose threshold of any chemical substance in a human being. This parameter is important to help predict the maximum recommended starting dose for the pharmaceuticals in clinical trial phase I. If the MTD is less than or equal to 0.477 then it is a lowly toxic compound. The MTD value of all the proposed CYP3A5 inhibitor molecules was found to be less than 0.376, which certainly suggests that all the molecules possess a toxic dose within the threshold. The skin sensitization (SS) of any pharmaceutical product indicates the adverse effect it will have on the skin after its use. This parameter evaluates any skin allergy after consumption of the molecules. Table 3 clearly shows that none of the molecules contained any kind of SS-positive effects on the human skin. Overall, from the above data, there is no space for any doubt about whether the proposed molecules may be toxic in nature after administration to the human body.

### 2.6. Molecular Dynamics Simulation

MD simulation is a pivotal computational technique for exploring the mobility of the macro-molecules in a user-defined aqueous medium. Five finally proposed CYP3A5 inhibitor molecules and RIT bound with CYP3A5 were considered for a 100ns MD simulation study. After the successful completion of the MD simulation, several parameters—including the protein backbone RMSD, ligand RMSD, root-mean square fluctuation (RMSF), radius of gyration (RoG), and the number of hydrogen bonds between protein and ligand—were evaluated and recorded. The maximum, minimum, and average values of the protein backbone RMSD, ligand RMSD, RMSF, and RoG are given in Table 4.

#### 2.6.1. Root-Mean-Square Deviation (RMSD)

##### Protein Backbone RMSD

**Figure 9:**
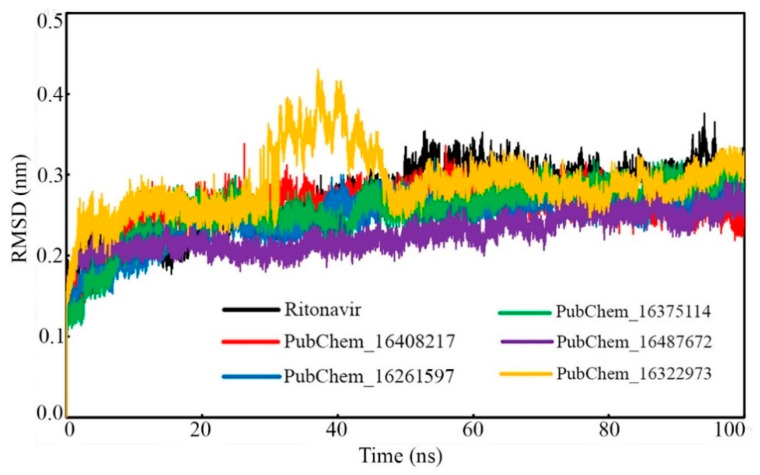
CYP3A5 backbone RMSD bound with CYP3A5 inhibitor molecules.

The protein backbone RMSDs of CYP3A5 bound with the proposed molecules and RIT were calculated and plotted against the time of simulation, and the plot is depicted in Figure 9. It was observed that all the protein–ligand systems remained consistent and were equilibrated very quickly from the initiation of the simulation to the end of the simulation. No significant deviation of the CYP3A5 backbone was observed in the dynamic states. In the case of the CYP3A5 backbone bound with PubChem_16322973, it was found to deviate slightly from 35 ns to 50ns. Afterward, the backbone remained consistent, similar to the other complexes, and maintained the same state until the end of the simulation. The potential deviation above might have been due to the higher fluctuation of the small molecules inside the active site. The average CYP3A5 backbone RMSD was found to be 0.269, 0.258, 0.247, 0.256, 0.227, and 0.288 nm when bound with RIT, PubChem_16408217, PubChem_16261597, PubChem_16375114, PubChem_16487672, and PubChem_16322973, respectively. Hence, the low average RMSD along with the consistent deviation of the CYP3A5 backbone clearly suggested the stability of the protein–ligand complexes in the dynamic state.

##### Ligand RMSD

**Figure 10:**
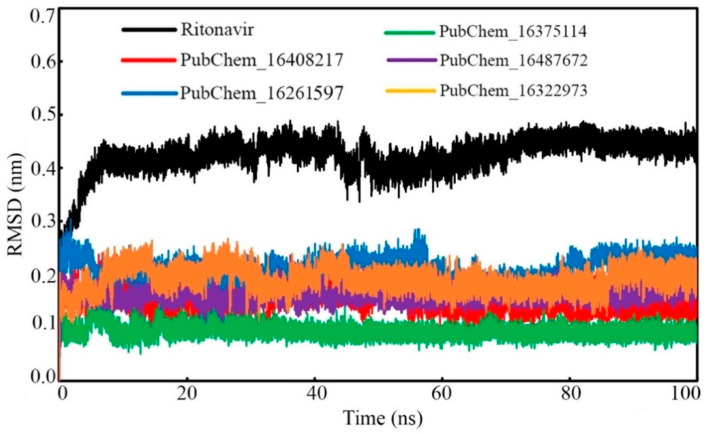
RMSD of proposed CYP3A5 molecules and RIT.

The ligand RMSD of each frame was plotted against the time of simulation, and this is given in Figure 10. All the molecules were found to deviate in a similar fashion almost from the beginning to the end of the simulation. PubChem_16375114 was found to be equilibrated with the lowest RMSD values. The rest of the proposed molecules were found to oscillate with almost similar RMSD values. It is important to note that not a single frame of all the molecules was found to deviate more than 0.304 nm. From Figure 10, it can be observed that the standard compound RIT was equilibrated at higher RMSD values in comparison to the proposed molecules. In the beginning, the RMSD of RIT was increased; at around 0.42 ns, it became consistent until the end of the simulation. Overall, from Table 4 and Figure 10, it is evident that all the small molecules oscillated inside the receptor cavity of CYP3A5 with smaller RMSD values.

#### 2.6.2. Root-Mean-Square Fluctuation (RMSF)

**Figure 11:**
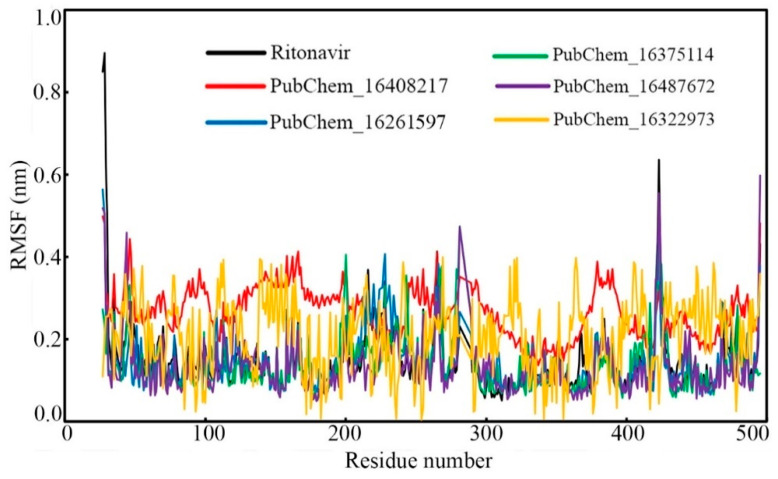
RMSF of individual amino acids of CYP3A5 bound with proposed molecules and RIT.

Fluctuations in the backbone of an individual amino acid residue play a critical role in maintaining the stability of the protein–ligand complex in the dynamic state. To check the fluctuation of every amino acid of CYP3A5 bound with the proposed molecules and RIT, the RMSF value was calculated, and it is displayed in Figure 11. Except for the amino acids of CYP3A5 bound with PubChem_16322973, the protein RMSF values bound with other molecules were found to fluctuate in an almost similar manner. Although the CYP3A5 amino residues bound with PubChem_16322973 fluctuate slight differently, not a single amino residue was found to deviate more than 0.400 nm. The differences between the maximum and average RMSF were found to be 0.742, 0.232, 0.417, 0.336, 0.465, and 0.190 nm when bound with the compounds RIT, PubChem_16408217, PubChem_16261597, PubChem_16375114, PubChem_16487672, and PubChem_16322973, respectively. The low RMSF values and fewer fluctuations in the RMSF values undoubtedly explain that the amino acids of CYP3A5 successfully held their native conformational state and held all the proposed small molecules tightly during the span of the MD simulation.

#### 2.6.3. Radius of Gyration (RoG)

**Figure 12:**
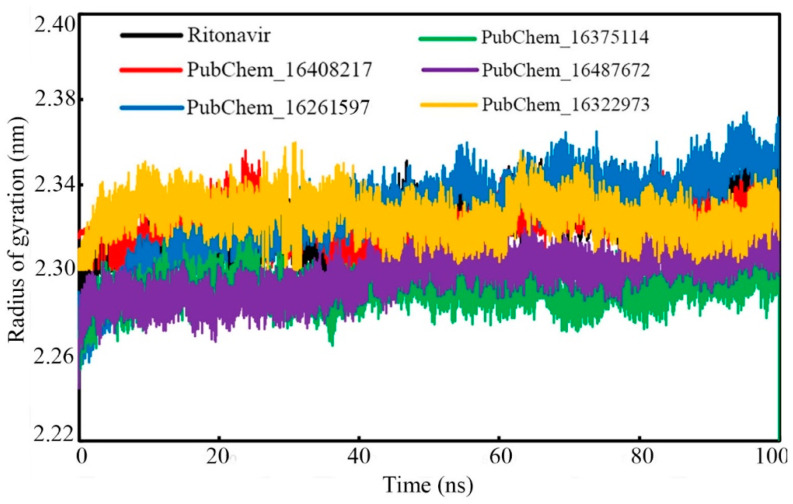
RoG of CYP3A5 bound with CYP3A5 molecules.

The rigidity of the molecular systems in dynamic states can be assessed through the RoG parameter. In the MD simulation, the folding of the protein molecule can be substantiated by an almost intact RoG variation. Frequent deviations of the RoG value indicate the unfolding nature of any protein molecule under study. In the present study, the RoG value of the CYP3A5 protein backbone with respect to the time of simulation was calculated and it is given in Figure 12. From the RoG values, it can be observed that mostly all the studied systems remained intact throughout the simulation’s duration. No significant deviation was observed in the RoG values. It is also important to note that the maximum value of RoG was found to be 2.374 nm for PubChem_16261597, and the minimum value was observed to be 2.205 nm for PubChem_16375114. The above data explained that for all the complexes, the RoG was found to vary within 0.169 nm. The average RoG values were found to be 2.322, 2.323, 2.327, 2.291, 2.297, and 2.235 nm for RIT, PubChem_16408217, PubChem_16261597, PubChem_16375114, PubChem_16487672, and PubChem_16322973, respectively. The close average values of all the complexes also corroborates the intactness of the system.

#### 2.6.4. Hydrogen Bond Analysis

**Figure 13:**
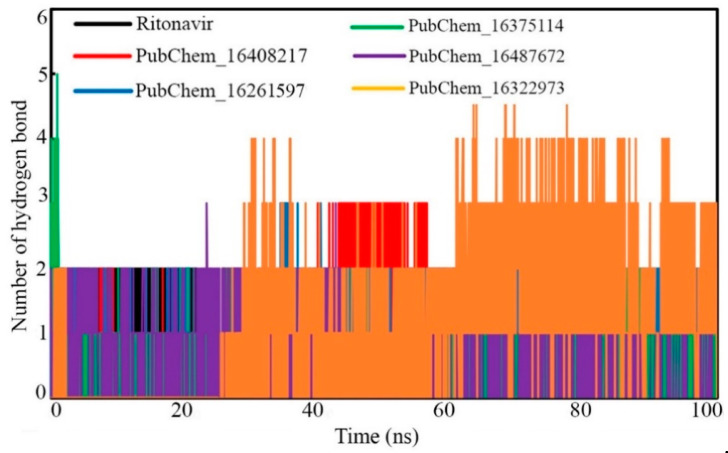
Number of hydrogen bonds vs. time of simulation.

Hydrogen bonding kinetics are critical for measuring the protein–ligand interaction’s stability in the simulation state. During the dynamic simulation, due to the rearrangement of the conformational integrity either in the protein or ligand atoms, there may be a potential for the breaking down and re-forming of HBs. Therefore, along with other non-binding interactions, the presence of HB interaction networks was evaluated for all the studied protein–ligand systems and was plotted against the simulation’ duration (Figure 13). A close observation suggests that out of a total of 50,000 frames, most of the frames either retained old HB(s) or formed new HB(s). It was found for all the molecules that very few frames were found without any HBs. It can be assumed that non-binding interactions, such as hydrophobic or van der Waals’ contacts, might have held the proposed molecules with CYP3A5 in the absence of HB(s).

### 2.7. Binding Free Energy Using MM-GBSA Approach

**Figure 14:**
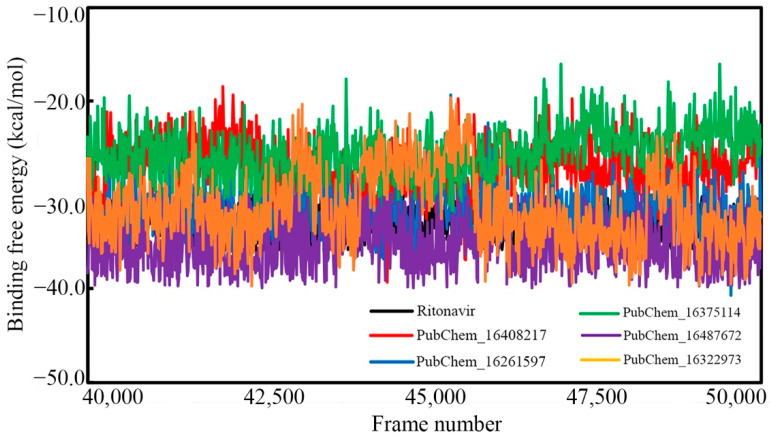
Binding free energy of CYP3A5 molecules vs. frame number.

In comparison to the conventional binding energy derived in molecular docking studies, the MM-GBSA-based binding free energy (ΔG_bind_) from MD simulation trajectories is believed to be more authenticated and reliable. The MM-GBSA method was used to calculate the ΔG_bind_ by utilizing the information of the last 20 ns or last 10,000 frames of each molecule bound with CYP3A5. Table 5 shows the average ΔG_bind_ and standard deviation values calculated for each proposed CYP3A5 inhibitor molecule. Moreover, the ΔG_bind_ value of each frame is given in Figure 14.

In particular, the ΔG_bind_ of RIT was found to be −33.015 (±1.578) kcal/mol. The ΔG_bind_ values of PubChem_16408217, PubChem_16261597, PubChem_16375114, PubChem_16487672, and PubChem_16322973 were found to be −27.165 (±2.840), −31.826 (±2.437), −25.557 (±2.617), −35.386 (±2.633), and −31.378 (±3.918) kcal/mol, respectively. Indeed, it is interesting to observe that all the proposed molecules were found to have higher or comparable ΔG_bind_ when tying the CYP3A5 as compared to RIT. It can be also observed from Figure 14 that the binding free energy of all the molecules varied between −40.700 to −16.028 kcal/mol. It is also important to note that not a single frame was found to have a positive ΔG_bind_ value, which suggests the occurrence of spontaneous molecular interactions during simulation. Hence, from the above observations, it can be postulated that the final molecules possess strong affections towards the CYP3A5 for exhibiting the necessary biological actions to inhibit the role of CYP3A5 in the cellular environment.

**Figure 15:**
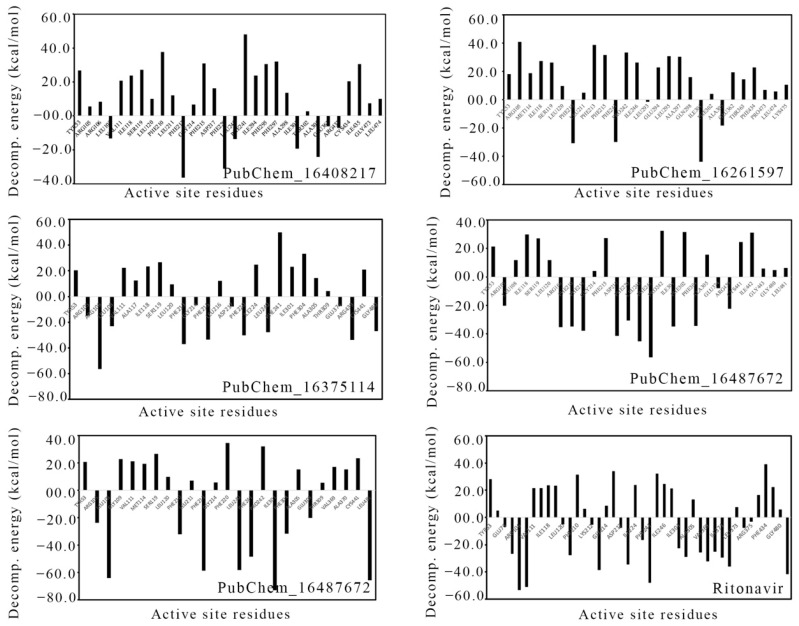
Per-residue decomposition energy of CYP3A5 amino acids around 5 Å of bound ligand.

The energy contribution of the amino acids present around 5 Å of the bound ligand was calculated through MM-GBSA approach. Likewise, for the ∆G_bind_ calculation, the last 20 ns or last 10,000 frames were used to calculate the per-residue decomposition energy, which is given in Figure 15. The high negative decomposition energy explained the increased tendency of the amino acid to bind with the ligand. Several key amino acids were found to have negative decomposition energy. It is expected that the ligand-binding amino acids in molecular docking should have a negative decomposition that shows an affinity towards the ligand. On close observation, it was found that almost all the ligand-binding amino acids possessed negative binding energies in the range of −7 to −66 kcal/mol. Moreover, a number of amino acids were found to have negative decomposition energies, but they failed to form any binding interactions in the molecular docking study. The above observation indicated that the amino acids with negative decomposition energies might have had the potential to interact with the ligands, but this failed due to the distance of the bond formation groups, which were out of range. Hence, the above decomposition energy from the MD simulation data undoubtedly explained the strong bonding between CYP3A5 and the ligands.

## 3. Materials and Methods

Virtual screening is a pivotal and widely used approach for computational chemists to explore the potential chemical agents that inhibit or activate macromolecular targets. Application of both receptor- and ligand-based approaches in the VS have been proved to be effective and successful strategies in drug-discovery research [51,52,53,54]. In the current study, molecular docking, NIB screening, ML-based screening, in silico pharmacokinetics, and toxicity assessments followed by MD simulation and binding free energy calculation using the MM-GBSA method were used to explore the promising new chemical entities as CYP3A5 modulators for therapeutic applications in cardiovascular diseases. All of the above approaches have already been proven to be effective and pioneering methodologies for the identification of lead-like molecules [4,9,55,56,57,58,59,60,61,62]. List of tools, approaches, and their purposes used in the current study are given in Table S1 (Supplementary File).

### 3.1. Ligand Preparation

The entire PubChem database [63], consisting of about 99 million compounds, was collected in the month of October 2020. The database was curated by implementing several criteria, including molecular weight (MW) of 160 to 500; molecular refractivity value of 40 to 300; hydrogen bond (HB) accepters and HB donors less than or equal to 10 and 5, respectively; number of rotatable bonds and number of atoms that is not more than 10 and 150, respectively; and hydrophobicity (logP) less than or equal to 5. The above criteria were implemented in the in-house python code to screen the molecules. After screening the entire database through the above criteria, a total of about 43 million compounds remained. With the help of the OpenBabel [64] tool, the above molecules were further considered to convert into a 3D format at the physiological pH (7.4) [65]. Further, the Gasteiger charge [66] was added to each molecule and converted into .pdbqt for the molecular docking in ADV [16].

### 3.2. Protein Preparation

The RCSB-Protein Databank (PDB) [67] is the largest repository for the 3D crystal coordinates of macromolecules. More than 186000 3D structures of the macromolecules generated by different experimental methods including x-ray, NMR, electron microscopy, and multi-methods are available (https://www.rcsb.org/stats, accessed on 20 December 2021). The 3D crystal structure of CYP3A5 with PDB ID: 5VEU [68] was collected from the RCSB-PDB. In order to consider the particular PDB ID, a number of criteria were considered, including atomic resolution, R-value, and date of deposition. The resolution and R-value of the selected CYP3A5 crystal structure were found to be 2.910 Å and 0.258 Å, respectively. A total of 480 amino acids were found in CYP3A5. Ritonavir (RIT), a human immunodeficiency virus type I protease inhibitor, was co-crystallized with CYP3A5. Before its use in the molecular docking procedure, the crystal CYP3A5 was prepared using the Autodock Tools (ADT) [15]. The missing atoms and amino acids were repaired followed by the crystal water molecules’ deletion and the addition of polar hydrogen. The Gasteiger charge [66] was added to the molecules. The atoms of the molecules were assigned the AD4 (Autodock 4) type and finally saved using the .pdbqt format. It is important to note that the transmembrane part of the CYP3A5 is reported as residue numbers 3 to 23 at the N-terminal. During the crystallization, the transmembrane portion of the CYP3A5, residue numbers 24–26, and five residues from the C-terminal were removed. Hence, the amino acid sequences are numbered from 27 to 495 [68]. The HEME and RIT are numbered as 601 and 602, respectively [68].

### 3.3. Molecular Docking

Molecular docking is one of the fundamental techniques of SBVS and is widely applied in the screening of large chemical databases within a shorter time span. This approach is fit for small molecules in the active site of the macromolecule in their potentially active conformation. The best pose (or conformation) of each molecule is identified through conformational analysis. Moreover, on the docking of any small molecule in the target site, along with the best pose, the binding energy is also estimated. Based on the binding energy, the multiple docked molecules can be ranked to segregate active and inactive compounds. In the current study, the essence of molecular docking was used to screen the curated PubChem database through ADV [16]. ADT is a freely available ready-to-use molecular docking program, and it is widely accepted and recommended by the scientific community. This comprehensive, intuitive, and excellent molecular docking tool is based on a simple scoring function and rapid-gradient-optimization conformational search.

The validation of the molecular docking protocol is required before it is used to screen any chemical compounds dataset. Self-docking [69] is one of the crucial docking protocol validation methods in which the co-crystal bound ligand is re-drawn and docked at the same position where the co-crystal ligand is bound. The main objective of the self-docking validation method is to check whether the molecular docking yields a comparable molecular conformation to the x-ray crystalized orientation [70]. The same approach was used in the current study, i.e., the structure of RIT was drawn in MarvinSketch (https://chemaxon.com/products/marvin, accessed on 21 December 2021) and docked at the active site of CYP3A5. The coordinate of co-crystal RIT was selected as an active site center. The grid size was finalized by manually inspecting the entire co-crystal RIT embedded inside the grid. Hence, the grid coordinates were selected as (−11.748, −49.503, and 24.963) along the x-, y-, and z-axes, respectively. The grid size was selected as 50 × 50 × 50. It is illustrated that the root-mean-square deviation (RMSD) ≤ 2 Å between co-crystal ligand and its best-docked pose validates the molecular docking protocol. The Lamda function [71] of AWS server was used to dock the entire dataset in the CYP3A5. It is an excellent, fast, and powerful application of Amazon Web Services [72] that runs as an event-driven, serverless platform. In general, it runs the code in response to events and automatically accomplishes the computing resources required by that code. On completion of the docking of the entire dataset, the binding energy of each molecule was explored. Initially, the binding energy of RIT was considered as a threshold to screen the molecules, and it was found that about 90% of docked molecules satisfied the threshold. Hence, to reduce the chemical space and achieve higher binding affinities of the molecules, the user-defined threshold value was considered by reducing the binding free energy. Screening of the docked compounds using the user defined threshold value has been performed in a number of recent studies, including by our research group [73].

### 3.4. Negative Image-Based (NIB) Screening

The NIB model is the formation of a negative image from the receptor cavity of any protein target. In this approach, the shape and electrostatic complementarity between the small molecule and target cavity are considered. The PANTHER program [74] is the program used to generate the NIB model by considering the shape and charge features of the protein cavity. During the NIB model’s development, the PANTHER program takes into account explicit water molecules, cofactors, and ions [19]. Without any prior knowledge about target-specific active and inactive small molecules, it can generate the model solely from the protein molecule. In the current study, the crystal structure of CYP3A5 (PDB ID: 5VEU) was used to generate the NIB model followed by the screening of molecules that remained after molecular docking. The binding site coordinates used in molecular docking were also considered in NIB model generation as the center of the site (-cent). Further, the pocket center was generated around the co-crystal bound ligand, i.e., RIT, with the -bmp option. Through the -ldlim option of PANTHER, the volume of the NIB model was restricted at the active site cavity. The NIB model developed from the CYP3A5 was used to screen the molecules retained after binding energy-based screening in molecular docking. The ShaEP program [36] was used for a similarly based screening. Before the screening, the charge was added to the ligands followed by the generation of 3D conformers. The shape and electrostatic features of each of the ligands were compared with the template of the NIB model of CYP3A5 by applying an equal amount of weight to both electrostatic and shape values. RIT was also screened using a similar manner. The ShaEP score of RIT was used as a threshold to screen out considered ligands.

### 3.5. Machine Learning-(ML) Based Screening

Molecules retained after NIB-based screening were further used to screen out ligands using the six ML matrices including DT [40], LR [41], *k*-NN [42], RF [43], SVM [44], and GBM [45]. A set of 16 active molecules targeting CYP3A5 was collected from BindingDB and it was used to generate the decoy molecules through the Decoy generator in the DUD-E server. A total of 6341 decoy molecules were found and amalgamated with an active set of molecules to form the training set. The entire training set was used to generate the molecular descriptors using the PaDEL software tool. Through the Wilcoxon’s rank sum test (p < 0.05) test, out of more than 1144 descriptors, 492 were found to be significant for the study. The training and validated class level models showed no significant differences in McNemar’s test [75]. Five-fold cross-validation (CV) was performed, which optimized hyperparameters for all these models. Further, to predict whether the compounds were active or inactive, the trained models were applied to the test dataset (compounds taken from ChEMBL library). The confusion matrix (CM) considered the following parameters: implicit active or true positive (TP), inactive or true negative (TN), predicted as active or false positive (FP), and predicted as inactive or false negative (FN). Based on CM, various performance indices were calculated, such as precision, recall (or sensitivity), F-score, accuracy, and specificity using the following expressions.

(1) $$\mathrm{Confusion}\;\mathrm{matrix}\;(\mathrm{CM})=\left[\begin{array}{l} \mathrm{TP}\;\mathrm{FP}\\ \mathrm{FN}\;\mathrm{TN} \end{array}\right]$$

(2) $$\mathrm{Precision}=\frac{\mathrm{TP}}{\left(\mathrm{FP}+\mathrm{TP}\right)}$$

(3) $$\mathrm{Recall}\;(\mathrm{or}\;\mathrm{Sensitivity})=\frac{\mathrm{TP}}{\left(\mathrm{TP}+\mathrm{FN}\right)}$$

(4) $$F-\mathrm{score}=2\times \frac{\mathrm{TP}}{(2\times \mathrm{TP}+\mathrm{FP}+\mathrm{FN})}$$

(5) $$\mathrm{Accuracy}=\frac{\left(\mathrm{TP}+\mathrm{TN}\right)}{\left(\mathrm{TP}+\mathrm{TN}+\mathrm{FP}+\mathrm{FN}\right)}$$

(6) $$\mathrm{Specificity}=\frac{\mathrm{TN}}{\mathrm{FP}+\mathrm{TN}}$$

The receiver operating characteristics’ (ROC) area under the curve (AUC) values for the six classifiers were also compared. Molecules carried forward after the ShaEP-based screening were considered for segregation into active and inactive using all the ML models. Molecules found active in each model were collected and merged. The final set of all active molecules obtained from all six models was carefully checked and duplicates were deleted. Retained molecules were used for in silico pharmacokinetics and toxicity assessments.

### 3.6. In Silico Pharmacokinetics and Toxicity Assessment

A number of pharmacokinetics and toxicity parameters of the retained molecules after ML-based screening were calculated using the SwissADME [46] and pkCSM [47] tools. Both are freely available online tools and accept the simplified molecular input line entry system (SMILES) [76] format of the molecules. With the help of SwissADME, the solubility, gastrointestinal (GI) absorption, blood brain barrier (BBB), synthetic accessibility, number of heavy atoms (HA), number of aromatic heavy atoms (AHA), number of rotatable bonds (RB), number of hydrogen bond (HB) acceptors (HBA) and donors (HBD), molar refractivity (MR), and total polar surface area (TPSA) were calculated. The toxicity parameters included AMES toxicity, maximum tolerated dose (MTD), oral rat acute toxicity (ORAT) minnow toxicity, and skin sensitization (SS), which were obtained from the pkCSM. Each of the above pharmacokinetics and toxicity parameters were checked for all the molecules found after ML-based screening. Molecules that satisfied the acceptable value of each parameter were considered for further assessments.

### 3.7. Molecular Dynamics (MD) Simulation

The MD simulation examines and determines the time-dependent dynamic behavior of a molecular system. Each of the final proposed molecules and RIT bound with CYP3A5 were considered for a 100 ns MD simulation. GROningen MAchine for Chemical Simulations (GROMACS) v2021.2 [77,78] was used to perform the MD simulation. The CHARMM-GUI [79], a web-based system generation tool for MD simulations, was used to prepare the protein–ligand complex system in explicit water molecules. The topology of CYP3A5 was generated using the CHARMM36 [80] forcefield. The general AMBER force field2 (GAFF2) [81] forcefield was used to generate the topology of each of the small molecules including RIT. The rectangular box was considered for the simulation and each complex was centered in it. The minimum distance was maintained as 10 Å between the atoms of the protein–ligand complex and the box edge. The system was solvated using the transferable intermolecular potential with 3 points (TIP3P) [82] water model. Each of the systems was neutralized by the addition of the required number of Na^+^ and Cl^−^ ions. Further, the systems were minimized using the steepest-descent algorithm to address the close contacts or overlaps between the atoms. To equally distribute the water molecules and ions around the system, each of the systems were equilibrated through NVT (constant number of particles, volume, and temperature). Finally, the production of the individual system was conducted for a 100 ns time span with a 2 fs timestep. The MD simulation trajectories were used to calculate several parameters, including CYP3A5 backbone RMSD, ligand RMSD, root-mean-square fluctuation (RMSF), radius of gyration (RoG), and number of HBs between CYP3A5 and ligands in each frame.

### 3.8. Molecular Mechanics Generalized Born Surface Area- (MM-GBSA) Based Binding Energy Calculation

MM-GBSA is one of the most accurate and popular binding free energy (ΔG_bind_) estimations of small molecules from the MD simulation trajectories. It is also computationally efficient and considered to be a better estimation in comparison to the many scoring functions. From the MD simulation trajectories, the (ΔG_bind_) of each of the final CYP3A5 molecules and RIT were calculated using the gmx_MMPBSA module [83]. The following expressions were used to calculate the ΔG_bind_.

(7) $$\Delta G_{\mathrm{bind}}=\langle G_{\mathrm{complex}}\rangle \;-\;\langle G_{\mathrm{receptor}}\rangle \;-\;\langle G_{\mathrm{ligand}}\rangle$$

where G_complex_, G_receptor_, and G_ligand_ are the binding energies of protein–ligand complex, receptor, and ligand, respectively.

The ΔG_bind_ can also be expressed as

(8) $$\Delta G_{\mathrm{bind}}=\Delta H\;-\;T\Delta S$$

ΔH is the enthalpy of binding, whereas TΔS represents the conformational entropy after ligand binding. On the removal of the entropic term, the estimated value represents the effective free energy [84]. The effective free energy is sufficient to compare the relative binding energies of any small molecule.

Further, the ΔH can be split into the following individual terms

(9) $$\Delta H={\Delta E}_{\mathrm{MM}}+{\Delta G}_{\mathrm{sol}}$$

where ΔE_MM_ can be expressed as a summation of bonded and non-bonded terms as below.

(10) $${\Delta E}_{\mathrm{MM}}={\Delta E}_{\mathrm{bonded}}+{\Delta E}_{\mathrm{nonbonded}}$$

E_bonded_ represents the combination of three terms such as bond stretching, angle bending, and torsion angle. The ΔE_nonbonded_ is a combination of electrostatic and van der Waals’ terms. Both expressions are given below.

(11) $${\Delta E}_{\mathrm{bonded}}={\Delta E}_{\mathrm{bond}\_\mathrm{length}}+{\Delta E}_{\mathrm{angle}}+{\Delta E}_{\mathrm{dihedral}}$$

(12) $${\Delta E}_{\mathrm{nonbonded}}={\Delta E}_{\mathrm{ele}}+{\Delta E}_{\mathrm{vdW}}$$

The solvation energy (ΔG_sol_) for GB models can be calculated using only the polar constituent. Whereas, the nonpolar (NP) constituent is mostly thought to be proportional to the molecule’s total solvent accessible surface area (SASA), with a proportionality constant derived from experimental solvation energies of small nonpolar molecules [85,86]. Both solvation and non-polar energy terms are expressed as given below.

(13) $${\Delta G}_{\mathrm{sol}}={\Delta G}_{\mathrm{polar}}+{\Delta G}_{\mathrm{non}-\mathrm{polar}}={\Delta G}_{\mathrm{GB}}+{\Delta G}_{\mathrm{non}-\mathrm{polar}}$$

(14) $${\Delta G}_{\mathrm{non}-\mathrm{polar}}={\mathrm{NP}}_{\mathrm{TENSION}}+\Delta_{\mathrm{SASA}}+{\mathrm{NP}}_{\mathrm{OFFSET}}$$

Upon successful calculation of the ΔG_bind_ of each molecule, they were documented along with the standard deviation.

The amino acids present around the active site of any protein target play a significant role by holding the ligand. The exploration of the potentiality of amino acids towards the ligand can be explored through per-residue decomposition energy. The last 20 ns of MD simulation trajectory was used to calculate the per-residue decomposition energy of CYP3A5 amino acids around 5 Å from the final molecules [87].

## 4. Conclusions

To explore the potential CYP3A5-inhibitor molecules for use in cardiovascular diseases, extensive and multiple levels of virtual screening were performed. About 99 million compounds from the PubChem database were initially screened by molecular docking followed by NIB screening as well as ML and in silico pharmacokinetics and toxicity assessments. Finally, five potential molecules were found to have a strong binding affinity towards CYP3A5. All the molecules consisted of a wide range of pharmacophoric features that might be important for bonding interactions with the active site amino acids of CYP3A5. The binding interaction profile was also explained by the comparable bond formation to the co-crystal ligand RIT. The cavity features of the NIB model have also been found to fit all the molecules, with their appropriate orientations corroborating the importance of the complementary shape and electrostatic features of both the ligands and target site. The acceptable pharmacokinetic profile and non-carcinogenic nature indicate that all the molecules will be easy to deliver to the target site and safe for use. Several statistical parameters calculated from the MD simulation have also explained the consistency and rigidity of the combined CYP3A5 and the proposed molecular systems. The high negative binding energies of each molecule obtained from the MD simulation trajectories have also described the potentiality towards CYP3A5. The per-residue decomposition energy of the ligand-binding amino acids showed strong affinity towards the ligand. Hence, it is clear from the extensive computational studies and data that the final proposed molecules possess all of the characteristics of potent and safer CYP3A5 molecules, which was confirmed via experimental validation through a number of approaches including the thermal shift assay, kinetic simulation study, fluorescence cross-correlation spectroscopy, atomic force microscopy, etc.

## Figures and Tables

**Table 1:** Performance indices for 5-fold CV using different ML classifiers.

Classifier	Precision	Recall	F-Score	Accuracy	Specificity
SVM	0.83 ± 0.01	0.80 ± 0.02	0.78 ± 0.02	0.82 ± 0.01	0.81 ± 0.01
RF	0.74 ± 0.01	0.61 ± 0.01	0.57 ± 0.01	0.67 ± 0.01	0.74 ± 0.01
KNN	0.54 ± 0.01	0.51 ± 0.02	0.48 ± 0.02	0.52 ± 0.01	0.54 ± 0.01
GBM	0.84 ± 0.02	0.81 ± 0.02	0.76 ± 0.01	0.82 ± 0.01	0.83 ± 0.02
DT	0.73 ± 0.01	0.61 ± 0.01	0.54 ± 0.01	0.67 ± 0.01	0.71 ± 0.01
LR	0.69 ± 0.01	0.62 ± 0.02	0.52 ± 0.01	0.66 ± 0.01	0.68 ± 0.01

**Table 2:** Pharmacokinetic and drug similarity parameters of proposed CYP3A5 molecules.

Mols.	Sol	GI	BBB	SA	HA	AHA	RB	HBA	HBD	MR	TPSA
PubChem_16408217	Soluble	High	No	4.46	34	12	7	4	1	133.24	91.72
PubChem_16261597	Soluble	High	No	5.40	31	6	8	6	3	112.01	107.61
PubChem_16375114	Soluble	High	No	4.11	32	14	5	4	1	130.87	132.10
PubChem_16487672	Soluble	High	No	3.83	34	17	7	6	1	133.76	140.81
PubChem_16322973	Soluble	High	No	4.12	33	17	7	6	0	128.04	96.78

Sol—Solubility; GI—Gastrointestinal absorption; BBB—Blood-brain barrier absorption; SA—Synthetic accessibility; HA—Number of heavy atoms; AHA—Number of aromatic heavy atoms; RB: Number of rotatable bonds; HBA—Number of hydrogen bond acceptor; HBD—Number of hydrogen bond donors; MR—Molar refractivity; TPSA—Total polar surface area.

**Table 3:** Toxicity assessment of proposed CYP3A5 molecules.

Mols	AMES Toxicity	MTD (Human)	ORAT (LD_50_)	Minnow Toxicity	SS
PubChem_16408217	No	−0.695	2.758	3.037	No
PubChem_16261597	No	0.139	2.501	3.995	No
PubChem_16375114	No	−0.065	2.739	1.961	No
PubChem_16487672	No	−0.138	2.13	0.889	No
PubChem_16322973	No	0.375	2.622	−0.227	No

MTD—Maximum tolerated dose; ORAT—Oral rat acute toxicity; SS—Skin sensitization.

**Table 4:** Statistical parameters of CYP3A5 molecules obtained from MD simulation trajectories.

		Ritonavir	M1	M2	M3	M4	M5
**Backbone RMSD (nm)**	Min.	0.000	0.000	0.000	0.000	0.000	0.000
	Max.	0.376	0.338	0.321	0.322	0.297	0.430
	Average	0.269	0.258	0.247	0.256	0.227	0.288
**RMSF (nm)**	Min.	0.051	0.136	0.054	0.052	0.050	0.003
	Max.	0.895	0.498	0.563	0.473	0.598	0.398
	Average	0.150	0.266	0.146	0.137	0.133	0.208
**Ligand RMSD (nm)**	Min.	0.000	0.000	0.000	0.000	0.000	0.000
	Max.	0.489	0.248	0.304	0.190	0.234	0.267
	Average	0.423	0.151	0.211	0.094	0.162	0.191
**RoG (nm)**	Min.	2.256	2.300	2.254	2.205	2.245	2.300
	Max.	2.357	2.363	2.374	2.316	2.328	2.360
	Average	2.322	2.323	2.327	2.291	2.297	2.325

M1—PubChem_16408217; M2—PubChem_16261597; M3—PubChem_16375114; M4—PubChem_16487672; M5—PubChem_16322973; Min.—Minimum; Max.—Maximum.

**Table 5:** The binding free energy of CYP3A5 molecules through MM-GBSA approach.

Molecule	Total ΔG_bind_ (kcal/mol)	Standard Deviation
Ritonavir	−33.015	±1.578
PubChem_16408217	−27.165	±2.840
PubChem_16261597	−31.826	±2.437
PubChem_16375114	−25.557	±2.617
PubChem_16487672	−35.386	±2.633
PubChem_16322973	−31.378	±3.918

## Supplementary Materials

The following supporting information can be downloaded at: https://www.mdpi.com/article/10.3390/ijms23169374/s1. References [88,89,90,91] are cited in the supplementary materials.

## References

[1] Bonam S.R., Sekar M., Guntuku G.S., Nerella S.G., Pawar A.K.M., Challa S.R., Eswara G.K., Mettu S. (2021). Role of pharmaceutical sciences in future drug discovery. Futur. Drug Discov..

[2] Tanrikulu Y., Krüger B., Proschak E. (2013). The holistic integration of virtual screening in drug discovery. Drug Discov. Today.

[3] Graff D.E., Shakhnovich E.I., Coley C.W. (2021). Accelerating high-throughput virtual screening through molecular pool-based active learning. Chem. Sci..

[4] Walters W.P., Wang R. (2020). New trends in virtual screening. J. Chem. Inf. Model..

[5] Mohs R.C., Greig N.H. (2017). Drug discovery and development: Role of basic biological research. Alzheimer’s Dement. Transl. Res. Clin. Interv..

[6] de Souza Neto L.R., Moreira-Filho J.T., Neves B.J., Maidana R.L.B.R., Guimarães A.C.R., Furnham N., Andrade C.H., Silva F.P. (2020). In silico Strategies to Support Fragment-to-Lead Optimization in Drug Discovery. Front. Chem..

[7] Bhunia S.S., Saxena M., Saxena A.K. (2021). Ligand- and Structure-Based Virtual Screening in Drug Discovery. Topics in Medicinal Chemistry.

[8] Varela-Rial A., Majewski M., De Fabritiis G. (2022). Structure based virtual screening: Fast and slow. Wiley Interdiscip. Rev. Comput. Mol. Sci..

[9] Bender B.J., Gahbauer S., Luttens A., Lyu J., Webb C.M., Stein R.M., Fink E.A., Balius T.E., Carlsson J., Irwin J.J. (2021). A practical guide to large-scale docking. Nat. Protoc..

[10] Friesner R.A., Banks J.L., Murphy R.B., Halgren T.A., Klicic J.J., Mainz D.T., Repasky M.P., Knoll E.H., Shelley M., Perry J.K. (2004). Glide: A New Approach for Rapid, Accurate Docking and Scoring. 1. Method and Assessment of Docking Accuracy. J. Med. Chem..

[11] Venkatachalam C.M., Jiang X., Oldfield T., Waldman M. (2003). LigandFit: A novel method for the shape-directed rapid docking of ligands to protein active sites. J. Mol. Graph. Model..

[12] Rarey M., Kramer B., Lengauer T., Klebe G. (1996). A fast flexible docking method using an incremental construction algorithm. J. Mol. Biol..

[13] Cole J.C., Nissink J.W.M., Taylor R. (2005). Protein-ligand docking and virtual screening with GOLD. Virtual Screening in Drug Discovery.

[14] (2013). Molecular Operating Environment (MOE), 2013.08.

[15] Ahn Y., Jun Y. (2007). Measurement of pain-like response to various NICU stimulants for high-risk infants. Early Hum. Dev..

[16] Trott O., Olson A.J. (2010). AutoDock Vina: Improving the speed and accuracy of docking with a new scoring function, efficient optimization, and multithreading. J. Comput. Chem..

[17] Ye W.L., Shen C., Xiong G.L., Ding J.J., Lu A.P., Hou T.J., Cao D.S. (2020). Improving docking-based virtual screening ability by integrating multiple energy auxiliary terms from molecular docking scoring. J. Chem. Inf. Model..

[18] Eberhardt J., Santos-Martins D., Tillack A.F., Forli S. (2021). AutoDock Vina 1.2.0: New Docking Methods, Expanded Force Field, and Python Bindings. J. Chem. Inf. Model..

[19] Ahinko M., Kurkinen S.T., Niinivehmas S.P., Pentikäinen O.T., Postila P.A. (2019). A practical perspective: The effect of ligand conformers on the negative image-based screening. Int. J. Mol. Sci..

[20] McDonnell A.M., Dang C.H. (2013). Basic Review of the Cytochrome P450 System. J. Adv. Pract. Oncol..

[21] Hakkola J., Hukkanen J., Turpeinen M., Pelkonen O. (2020). Inhibition and induction of CYP enzymes in humans: An update. Arch. Toxicol..

[22] Williams J.A., Hyland R., Jones B.C., Smith D.A., Hurst S., Goosen T.C., Peterkin V., Koup J.R., Ball S.E. (2004). Drug-drug interactions for UDP-glucuronosyltransferase substrates: A pharmacokinetic explanation for typically observed low exposure (AUC 1/AUC) ratios. Drug Metab. Dispos..

[23] Wienkers L.C., Heath T.G. (2005). Predicting in vivo drug interactions from in vitro drug discovery data. Nat. Rev. Drug Discov..

[24] Danielson P.Á. (2005). The Cytochrome P450 Superfamily: Biochemistry, Evolution and Drug Metabolism in Humans. Curr. Drug Metab..

[25] Dennison J.B., Kulanthaivel P., Barbuch R.J., Renbarger J.L., Ehlhardt W.J., Hall S.D. (2006). Selective metabolism of vincristine in vitro by CYP3A5. Drug Metab. Dispos..

[26] Lu Y., Hendrix C.W., Bumpus N.N. (2012). Cytochrome P450 3A5 plays a prominent role in the oxidative metabolism of the anti-human immunodeficiency virus drug maraviroc. Drug Metab. Dispos..

[27] Khan A.R., Raza A., Firasat S., Abid A. (2020). CYP3A5 gene polymorphisms and their impact on dosage and trough concentration of tacrolimus among kidney transplant patients: A systematic review and meta-analysis. Pharm. J..

[28] Dai Y., Hebert M.F., Isoherranen N., Davis C.L., Marsh C., Shen D.D., Thummel K.E. (2006). Effect of CYP3A5 polymorphism on tacrolimus metabolic clearance in vitro. Drug Metab. Dispos..

[29] Patel J.K., Kobashigawa J.A. (2007). Tacrolimus in heart transplant recipients: An overview. BioDrugs.

[30] Zhang Y.P., Zuo X.C., Huang Z.J., Cai J.J., Wen J., Duan D.D., Yuan H. (2014). CYP3A5 polymorphism, amlodipine and hypertension. J. Hum. Hypertens..

[31] Wu J.J., Cao Y.F., Feng L., He Y.Q., Hong J.Y., Dou T.Y., Wang P., Hao D.C., Ge G.B., Yang L. (2017). A Naturally Occurring Isoform-Specific Probe for Highly Selective and Sensitive Detection of Human Cytochrome P450 3A5. J. Med. Chem..

[32] Niwa T., Narita K., Okamoto A., Murayama N., Yamazaki H. (2019). Comparison of steroid hormone hydroxylations by and docking to human cytochromes P450 3A4 and 3A5. J. Pharm. Pharm. Sci..

[33] Niwa T., Murayama N., Imagawa Y., Yamazaki H. (2017). Comparison of catalytic and inhibitory properties in human drug-metabolizing cytochrome P450 3A4 and 3A5 for various compounds including endogenous steroid hormones and azole antifungals by molecular docking simulation. Drug Metab. Pharmacokinet..

[34] Dai Z.R., Ning J., Sun G.B., Wang P., Zhang F., Ma H.Y., Zou L.W., Hou J., Wu J.J., Ge G.B. (2019). Cytochrome P450 3A enzymes are key contributors for hepatic metabolism of bufotalin, a natural constitute in Chinese medicine Chansu. Front. Pharmacol..

[35] Wang J., Buchman C.D., Seetharaman J., Miller D.J., Huber A.D., Wu J., Chai S.C., Garcia-Maldonado E., Wright W.C., Chenge J. (2021). Unraveling the Structural Basis of Selective Inhibition of Human Cytochrome P450 3A5. J. Am. Chem. Soc..

[36] Vainio M.J., Puranen J.S., Johnson M.S. (2009). ShaEP: Molecular overlay based on shape and electrostatic potential. J. Chem. Inf. Model..

[37] Gilson M.K., Liu T., Baitaluk M., Nicola G., Hwang L., Chong J. (2016). BindingDB in 2015: A public database for medicinal chemistry, computational chemistry and systems pharmacology. Nucleic Acids Res..

[38] Mysinger M.M., Carchia M., Irwin J.J., Shoichet B.K. (2012). Directory of useful decoys, enhanced (DUD-E): Better ligands and decoys for better benchmarking. J. Med. Chem..

[39] Yap C.W. (2011). PaDEL-descriptor: An open source software to calculate molecular descriptors and fingerprints. J. Comput. Chem..

[40] Quinlan J.R. (1987). Simplifying decision trees. Int. J. Man. Mach. Stud..

[41] Sperandei S. (2014). Understanding logistic regression analysis. Biochem. Medica.

[42] Altman N.S. (1992). An introduction to kernel and nearest-neighbor nonparametric regression. Am. Stat..

[43] Ho T.K. (1995). Random decision forests. Proceedings of the International Conference on Document Analysis and Recognition, ICDAR.

[44] Cortes C., Vapnik V. (1995). Support-Vector Networks. Mach. Learn..

[45] Friedman J.H. (2001). Greedy function approximation: A gradient boosting machine. Ann. Stat..

[46] Daina A., Michielin O., Zoete V. (2017). SwissADME: A free web tool to evaluate pharmacokinetics, drug-likeness and medicinal chemistry friendliness of small molecules. Sci. Rep..

[47] Pires D.E.V., Blundell T.L., Ascher D.B. (2015). pkCSM: Predicting small-molecule pharmacokinetic and toxicity properties using graph-based signatures. J. Med. Chem..

[48] Saba N., Bhuyan R., Nandy S., Seal A. (2015). Differential Interactions of Cytochrome P450 3A5 and 3A4 with Chemotherapeutic Agent-Vincristine: A Comparative Molecular Dynamics Study. Anticancer. Agents Med. Chem..

[49] Ghose A.K., Viswanadhan V.N., Wendoloski J.J. (1999). A knowledge-based approach in designing combinatorial or medicinal chemistry libraries for drug discovery. 1. A qualitative and quantitative characterization of known drug databases. J. Comb. Chem..

[50] Polêto M.D., Rusu V.H., Grisci B.I., Dorn M., Lins R.D., Verli H. (2018). Aromatic rings commonly used in medicinal chemistry: Force fields comparison and interactions with water toward the design of New Chemical Entities. Front. Pharmacol..

[51] Vázquez J., López M., Gibert E., Herrero E., Javier Luque F. (2020). Merging ligand-based and structure-based methods in drug discovery: An overview of combined virtual screening approaches. Molecules.

[52] Shoichet B.K. (2004). Virtual screening of chemical libraries. Nature.

[53] Schneider G. (2010). Virtual screening: An endless staircase?. Nat. Rev. Drug Discov..

[54] Westermaier Y., Barril X., Scapozza L. (2015). Virtual screening: An in silico tool for interlacing the chemical universe with the proteome. Methods.

[55] Liu X., Shi D., Zhou S., Liu H., Liu H., Yao X. (2018). Molecular dynamics simulations and novel drug discovery. Expert Opin. Drug Discov..

[56] Torres P.H.M., Sodero A.C.R., Jofily P., Silva-Jr F.P. (2019). Key topics in molecular docking for drug design. Int. J. Mol. Sci..

[57] Kontoyianni M. (2017). Docking and virtual screening in drug discovery. Methods in Molecular Biology.

[58] Jokinen E.M., Postila P.A., Ahinko M., Niinivehmas S., Pentikäinen O.T. (2019). Fragment- and negative image-based screening of phosphodiesterase 10A inhibitors. Chem. Biol. Drug Des..

[59] Adeshina Y.O., Deeds E.J., Karanicolas J. (2020). Machine learning classification can reduce false positives in structure-based virtual screening. Proc. Natl. Acad. Sci. USA.

[60] Gupta A., Zhou H.X. (2021). Machine Learning-Enabled Pipeline for Large-Scale Virtual Drug Screening. J. Chem. Inf. Model..

[61] Ekins S., Mestres J., Testa B. (2007). In silico pharmacology for drug discovery: Methods for virtual ligand screening and profiling. Br. J. Pharmacol..

[62] Rim K.T. (2020). In silico prediction of toxicity and its applications for chemicals at work. Toxicol. Environ. Health Sci..

[63] Kim S., Thiessen P.A., Bolton E.E., Chen J., Fu G., Gindulyte A., Han L., He J., He S., Shoemaker B.A. (2016). PubChem substance and compound databases. Nucleic Acids Res..

[64] O’Boyle N.M., Banck M., James C.A., Morley C., Vandermeersch T., Hutchison G.R. (2011). Open Babel: An Open chemical toolbox. J. Cheminform..

[65] Manallack D.T., Prankerd R.J., Yuriev E., Oprea T.I., Chalmers D.K. (2013). The significance of acid/base properties in drug discovery. Chem. Soc. Rev..

[66] Gasteiger J., Marsili M. (1978). A new model for calculating atomic charges in molecules. Tetrahedron Lett..

[67] Berman H., Henrick K., Nakamura H. (2003). Announcing the worldwide Protein Data Bank. Nat. Struct. Biol..

[68] Hsu M.H., Savas U., Johnson E.F. (2018). The X-ray crystal structure of the human mono-oxygenase cytochrome P450 3A5-ritonavir complex reveals active site differences between P450s 3A4 and 3A5. Mol. Pharmacol..

[69] Cross J.B., Thompson D.C., Rai B.K., Baber J.C., Fan K.Y., Hu Y., Humblet C. (2009). Comparison of several molecular docking programs: Pose prediction and virtual screening accuracy. J. Chem. Inf. Model..

[70] Erickson J.A., Jalaie M., Robertson D.H., Lewis R.A., Vieth M. (2004). Lessons in Molecular Recognition: The Effects of Ligand and Protein Flexibility on Molecular Docking Accuracy. J. Med. Chem..

[71] Mishra A. (2019). AWS Lambda. Machine Learning in the AWS Cloud.

[72] Al-Sayyed R.M.H., Hijawi W.A., Bashiti A.M., Al Jarah I., Obeid N., Adwan O.Y. (2019). An Investigation of Microsoft Azure and Amazon web services from users’ perspectives. Int. J. Emerg. Technol. Learn..

[73] Islam M.A., Subramanyam Rallabandi V.P., Mohammed S., Srinivasan S., Natarajan S., Dudekula D.B., Park J. (2021). Screening of β1-and β2-adrenergic receptor modulators through advanced pharmacoinformatics and machine learning approaches. Int. J. Mol. Sci..

[74] Niinivehmas S.P., Salokas K., Lätti S., Raunio H., Pentikäinen O.T. (2015). Ultrafast protein structure-based virtual screening with Panther. J. Comput. Aided. Mol. Des..

[75] McNemar Q. (1947). Note on the sampling error of the difference between correlated proportions or percentages. Psychometrika.

[76] Weininger D. (1988). SMILES, a Chemical Language and Information System: 1: Introduction to Methodology and Encoding Rules. J. Chem. Inf. Comput. Sci..

[77] Abraham M.J., Murtola T., Schulz R., Páll S., Smith J.C., Hess B., Lindah E. (2015). Gromacs: High performance molecular simulations through multi-level parallelism from laptops to supercomputers. SoftwareX.

[78] Lindahl A., Hess VD S., van der Spoel D. (2020). GROMACS 2021.3 Source Code 2021. Zenodo April.

[79] Jo S., Kim T., Iyer V.G., Im W. (2008). Charmm-Gui: A web-based graphical user interface for CHARMM. J. Comput. Chem..

[80] Huang J., Rauscher S., Nawrocki G., Ran T., Feig M., De Groot B.L., Grubmüller H., MacKerell A.D. (2016). CHARMM36m: An improved force field for folded and intrinsically disordered proteins. Nat. Methods.

[81] He X., Man V.H., Yang W., Lee T.S., Wang J. (2020). A fast and high-quality charge model for the next generation general AMBER force field. J. Chem. Phys..

[82] Mark P., Nilsson L. (2001). Structure and dynamics of the TIP3P, SPC, and SPC/E water models at 298 K. J. Phys. Chem. A.

[83] Valdés-Tresanco M.S., Valdés-Tresanco M.E., Valiente P.A., Moreno E. (2021). Gmx_MMPBSA: A New Tool to Perform End-State Free Energy Calculations with GROMACS. J. Chem. Theory Comput..

[84] Wang E., Sun H., Wang J., Wang Z., Liu H., Zhang J.Z.H., Hou T. (2019). End-Point Binding Free Energy Calculation with MM/PBSA and MM/GBSA: Strategies and Applications in Drug Design. Chem. Rev..

[85] Sitkoff D., Sharp K.A., Honig B. (1994). Accurate calculation of hydration free energies using macroscopic solvent models. J. Phys. Chem..

[86] Tan C., Tan Y.H., Luo R. (2007). Implicit nonpolar solvent models. J. Phys. Chem. B.

[87] Gohlke H., Kiel C., Case D.A. (2003). Insights into protein-protein binding by binding free energy calculation and free energy decomposition for the Ras-Raf and Ras-RalGDS complexes. J. Mol. Biol..

[88] Berman H.M., Westbrook J., Feng Z., Gilliland G., Bhat T.N., Weissig H., Shindyalov I.N., Bourne P.E. (2000). The Protein Data Bank. Nucleic Acids Res..

[89] Landrum G. RDKit: Open-Source Cheminformatics Software. http://Www.Rdkit.Org/.

[90] Steffen C., Thomas K., Huniar U., Hellweg A., Rubner O., Schroer A. (2009). AutoDock4 and AutoDockTools4: Automated Docking with Selective Receptor Flexibility. J. Comput. Chem..

[B91-ijms-23-09374] 91.ChemAxon Marvin Sketch.

