# Supplementary material for: Identification of Potential Cytochrome P450 3A5 Inhibitors: An Extensive Virtual Screening through Molecular Docking, Negative Image-Based Screening, Machine Learning and Molecular Dynamics Simulation Studies
Source: Int J Mol Sci. 2022 Aug 19;23(16):9374. doi: 10.3390/ijms23169374 (PMC9409045; doi:10.3390/ijms23169374)
Supplement: Supplementary file 1 [file ijms-23-09374-s001.zip › ijms-1864732-supplementary.pdf]

Identification of potential Cytochrome P450 3A5 molecules: An extensive virtual screening through molecular docking, negative image-based screening, machine learning and molecular dynamics simulation studies

Supplementary data

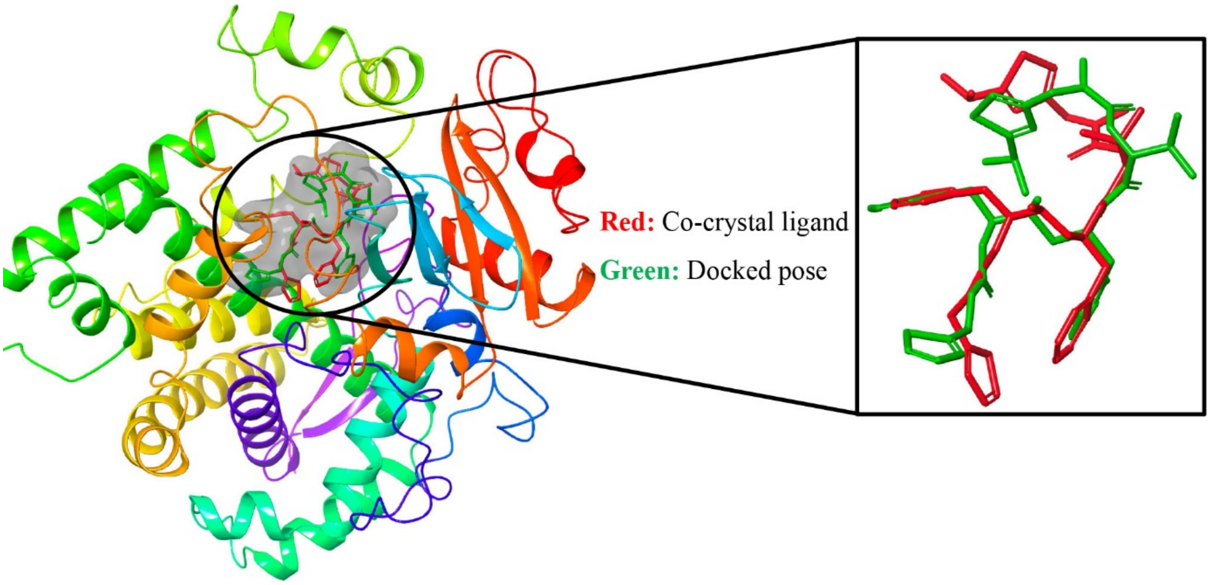

Figure S1. Superimposition of co-crystal ligand (RIT) and best docked pose of RIT

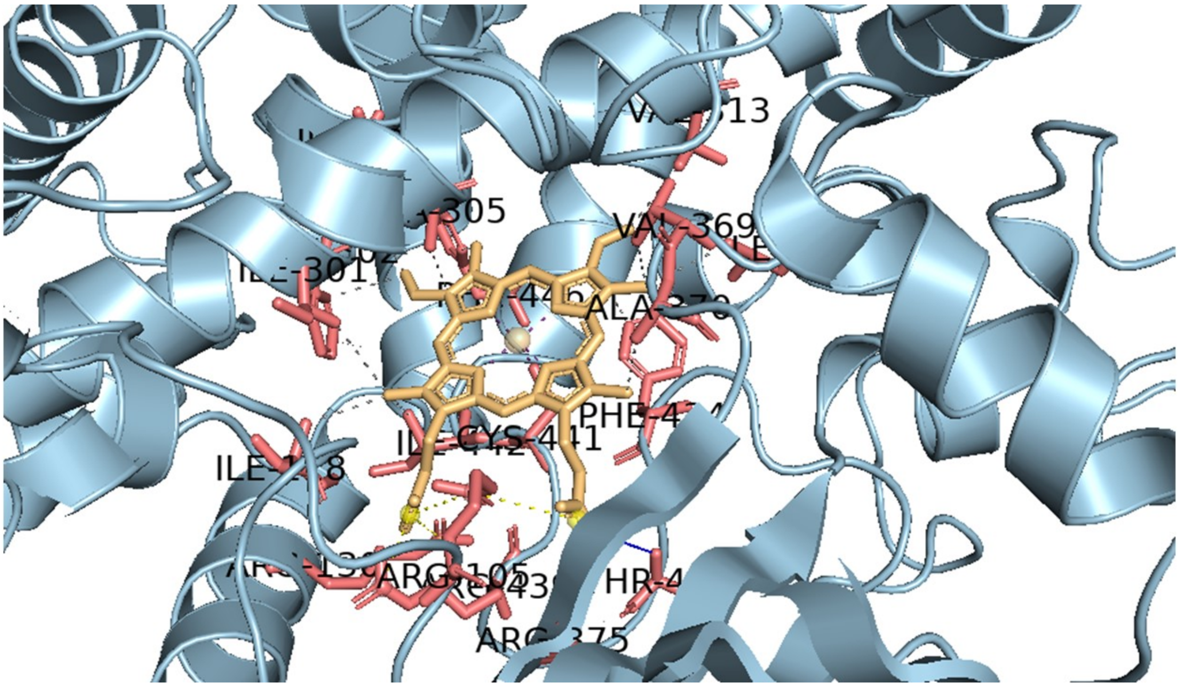

Figure S2. Binding interactions profile of HEME with CYP3A5

Table S1. Methodologies, purpose and tools used in the screening of PubChem database against CYP3A5

Methodology	Tool/method	Purpose
Database	1. PubChem[1]<br>2. Protein Data Bank[2]	The small molecule dataset obtained from the PubChem<br>To get the 3D coordinates of the CYP3A5
Screening of the molecules	PythonRDkit[3]	Screening of the molecular docking results<br>Screening and preparation of small molecules
Machine learning	scikit-learn package in Python	Development of machine learning models
Molecular docking and preparation	1. Autodock Vina[4]<br>2. Autodock tools[5]	Molecular docking between small molecules and CYP3A5<br>Preparation of the CYP3A5 3D structure
Small molecule Drawing	MarvinSketch[6]	2D structure generation of the small molecules
Negative image based modelling	PANTHER[7]	Negative image based model development
Screening	ShaEP[8]	Screening the molecules through negative image based models
Descriptors generation	PaDEL[9]	Generation of the molecular descriptors
Machine learning	1. Random Forest[10]<br>2. Support Vector Machine[11]<br>3. Gradient Boosting Machine[12]<br>4. k-nearest neighbourhood[13]<br>5. Decision Tree[14]<br>6. Logistic Regression[15]	Machine learning models were generated using each of approach and used for the screening of the small molecules
Pharmacokinetics	SwissADME[16]	Generation of pharmacokinetic parameters of final CYP3A5 molecules
Toxicity	pkCSM[17]	Toxicity assessment of final CYP3A5 molecules

MD Simulation	Gromacs[18]	Explore the dynamic behaviour of the small molecules inside the CYP3A5
Binding affinity	gmx_MMPBSA[19]	Explore the binding of affinity of the of final CYP3A5 molecules from the MD simulation trajectories.

References

1. Kim, S.; Thiessen, P.A.; Bolton, E.E.; Chen, J.; Fu, G.; Gindulyte, A.; Han, L.; He, J.; He, S.; Shoemaker, B.A.; et al. PubChem substance and compound databases. *Nucleic Acids Res.* **2016**, *44*, D1202–D1213, doi:10.1093/nar/gkv951.
2. Berman, H.M.; Westbrook, J.; Feng, Z.; Gilliland, G.; Bhat, T.N.; Weissig, H.; Shindyalov, I.N.; Bourne, P.E. The Protein Data Bank. *Nucleic Acids Res.* **2000**, *28*, 235–242.
3. Landrum, G. RDKit: Open-Source Cheminformatics Software. *Http://Www.Rdkit.Org/* **2021**.
4. Trott, O.; Olson, A.J. AutoDock Vina: Improving the speed and accuracy of docking with a new scoring function, efficient optimization, and multithreading. *J. Comput. Chem.* **2009**, NA-NA, doi:10.1002/jcc.21334.
5. Steffen, C.; Thomas, K.; Huniar, U.; Hellweg, A.; Rubner, O.; Schroer, A. AutoDock4 and AutoDockTools4: Automated Docking with Selective Receptor Flexibility. *J. Comput. Chem.* **2010**.
6. ChemAxon Marvin Sketch.
7. Niinivehmas, S.P.; Salokas, K.; Lätti, S.; Raunio, H.; Pentikäinen, O.T. Ultrafast protein structure-based virtual screening with Panther. *J. Comput. Aided. Mol. Des.* **2015**, *29*, 989–1006, doi:10.1007/s10822-015-9870-3.
8. Vainio, M.J.; Puranen, J.S.; Johnson, M.S. ShaEP: Molecular overlay based on shape and electrostatic potential. *J. Chem. Inf. Model.* **2009**, *49*, 492–502, doi:10.1021/ci800315d.
9. Yap, C.W. PaDEL-descriptor: An open source software to calculate molecular descriptors and fingerprints. *J. Comput. Chem.* **2011**, *32*, 1466–1474, doi:10.1002/jcc.21707.
10. Ho, T.K. Random decision forests. In Proceedings of the Proceedings of the International Conference on Document Analysis and Recognition, ICDAR; 1995; Vol. 1, pp. 278–282.
11. Cortes, C.; Vapnik, V. Support-Vector Networks. *Mach. Learn.* **1995**, *20*, 273–297, doi:10.1023/A:1022627411411.
12. Friedman, J.H. Greedy function approximation: A gradient boosting machine. *Ann. Stat.* **2001**, *29*, 1189–1232, doi:10.1214/aos/1013203451.
13. Altman, N.S. An introduction to kernel and nearest-neighbor nonparametric regression. *Am. Stat.* **1992**, *46*, 175–185, doi:10.1080/00031305.1992.10475879.
14. Quinlan, J.R. Simplifying decision trees. *Int. J. Man. Mach. Stud.* **1987**, *27*, 221–234, doi:10.1016/S0020-7373(87)80053-6.
15. Sperandei, S. Understanding logistic regression analysis. *Biochem. Medica* **2014**, *24*, 12–18, doi:10.11613/BM.2014.003.
16. Daina, A.; Michielin, O.; Zoete, V. SwissADME: A free web tool to evaluate pharmacokinetics, drug-likeness and medicinal chemistry friendliness of small molecules. *Sci. Rep.* **2017**, *7*, doi:10.1038/srep42717.
17. Pires, D.E.V.; Blundell, T.L.; Ascher, D.B. pkCSM: Predicting small-molecule pharmacokinetic and toxicity properties using graph-based signatures. *J. Med. Chem.*

2015, *58*, 4066–4072, doi:10.1021/acs.jmedchem.5b00104.
18. Lindahl; Abraham; Hess; van der Spoel GROMACS 2021.3 Source code **2021**.
19. Valdés-Tresanco, M.S.; Valdés-Tresanco, M.E.; Valiente, P.A.; Moreno, E. Gmx_MMPBSA: A New Tool to Perform End-State Free Energy Calculations with GROMACS. *J. Chem. Theory Comput.* **2021**, *17*, 6281–6291, doi:10.1021/acs.jctc.1c00645.
